# Endoplasmic reticulum stress exacerbates ischemia-reperfusion-induced pulmonary endothelial barrier dysfunction by activating TXNDC5

**DOI:** 10.1016/j.jare.2025.12.039

**Published:** 2025-12-26

**Authors:** Yu Wang, Pu Chen, Shiqian Huang, Lin Chen, Yangyang Ge, Heng Gu, Yangqi Chu, Yiyi Yang, Yun Lin, Shanglong Yao

**Affiliations:** a Department of Anesthesiology, Union Hospital, Tongji Medical College, Huazhong University of Science and Technology, Wuhan 430022, China; b Institute of Anesthesia and Critical Care Medicine, Union Hospital, Tongji Medical College, Huazhong University of Science and Technology, Wuhan 430022, China; c Key Laboratory of Anesthesiology and Resuscitation (Huazhong University of Science and Technology), Ministry of Education, China

**Keywords:** Lung ischemia–reperfusion injury, TXNDC5, Endothelial dysfunction, Endoplasmic reticulum stress, ATF6, eNOS

## Abstract

**Introduction:**

Lung ischemia–reperfusion injury (LIRI)—a fatal complication following major surgery, trauma, or shock— is characterized by pulmonary endothelial barrier (PEB) dysfunction. Although thioredoxin domain-containing protein 5 (TXNDC5) regulates endothelial homeostasis, its role in LIRI pathogenesis remains unknown.

**Objectives:**

In the present study, we investigate the role of TXNDC5 in LIRI-induced PEB disruption and explore its underlying mechanisms and therapeutic potential.

**Methods:**

We established LIRI models in rats (left pulmonary hilum occlusion) and human umbilical vein endothelial cells (oxygen-glucose deprivation/reperfusion). TXNDC5 expression was modulated via AAV-shRNA knockdown, whereas endoplasmic reticulum (ER) stress was inhibited pharmacologically (4-PBA and Ceapin-A7). Structural and functional changes of the PEB, including HSP90/eNOS complex stability, were examined. Mechanistic studies included ChIP-seq identified ATF6 binding to the TXNDC5 promoter, followed by rescue experiments with ATF6 inhibition and TXNDC5 overexpression. Clinical validation was performed using plasma proteomics in patients with LIRI. A TXNDC5-based nomogram model for LIRI early-warning was constructed and internally validated. Incremental prediction value of TXNDC5 was evaluated using net reclassification improvement (NRI) and integrated discrimination improvement (IDI).

**Results:**

Single-cell RNA sequencing revealed TXNDC5 upregulation in pulmonary endothelial cells during LIRI, correlating with PEB disruption, apoptosis, ER stress, and NF-κB activation. TXNDC5 knockdown restored HSP90/eNOS stability and improved endothelial integrity *in vitro*/*vivo*. Ceapin-A7, or 4-PBA, attenuated TXNDC5-driven PEB injury, and TXNDC5 overexpression confirmed the causal role of the ATF6-TXNDC5 axis. Mechanistically, ER stress promoted TXNDC5 transcription via ATF6 binding to its promoter. Clinically, elevated plasma TXNDC5 levels predicted LIRI and correlated with the severity of the condition. Incorporating the TXNDC5 index into the clinical predictors model enhanced the forecast of LIRI, as demonstrated by the NRI and IDI.

**Conclusion:**

Our findings demonstrate that ER stress-induced TXNDC5 upregulation exacerbates LIRI by impairing HSP90/eNOS-dependent endothelial function. ER stress-TXNDC5 signaling is a mechanistically distinct and potential target in LIRI.

## Highlights

• TXNDC5 serves dual roles as both a pathological mediator and clinical biomarker in lung ischemia-reperfusion injury (LIRI).
• ATF6-dependent endoplasmic reticulum stress triggers TXNDC5 transcription, leading to LIRI.
• TXNDC5 impairs endothelial function by disrupting HSP90/eNOS signaling pathways.
• The TXNDC5-based predictive model shows excellent LIRI diagnostic accuracy (AUC=0.822).

## Graphical abstract

**Figure:**
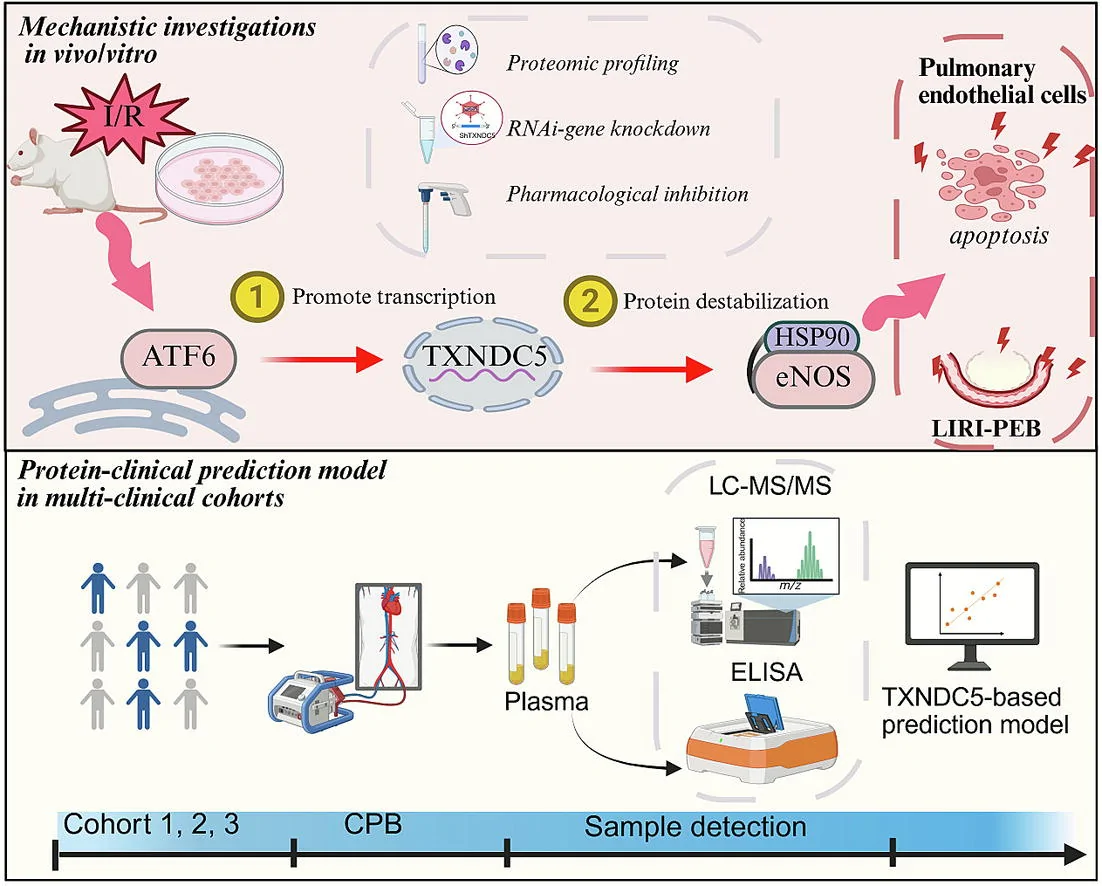

## Introduction

Ischemia/reperfusion (I/R)-induced acute lung injury (ALI/ARDS) causes pulmonary vascular injury, interstitial edema, and inflammation, leading to severe hypoxia and acute respiratory failure [1]. The disease evolves through three phases—exudative, proliferative, and fibrotic—with pulmonary endothelial barrier (PEB) disruption dominating the early exudative phase. Such PEB breakdown drives capillary leakage, edema formation, and amplified inflammatory injury, contributing to morbidity and mortality [[2], [3], [4]]. Although multiple pathogenic mechanisms (e.g., oxidative stress, endoplasmic reticulum (ER) stress, and apoptosis) have been implicated in I/R-induced PEB damage [[5], [6], [7], [8]], no clinically actionable protein targets currently exist, underscoring the need for targeted therapeutic strategies [9].

Thioredoxin domain-containing 5 (TXNDC5), an ER-localized protein disulfide isomerase (PDI), regulates disulfide bond formation and rearrangement [10]. Under conditions such as hypoxia and reperfusion, endothelial cells exhibit aberrant TXNDC5 activation [11]; hence, TXNDC5 is referred to as endothelial PDI. While its role in chronic cardiovascular, inflammatory, and fibrotic processes is established [[12], [13], [14], [15]], its function in lung injury, particularly in the context of I/R, is entirely uncharacterized. Although we previously observed TXNDC5 upregulation in patients with lung ischemia-reperfusion injury (LIRI) and rat models [16], and others have linked it to eNOS destabilization in atherosclerosis [14], its specific role in LIRI-induced endothelial barrier dysfunction remains unknown.

As an ER-resident PDI, TXNDC5 facilitates protein folding and participates in the unfolded protein response (UPR) during ER stress [10,14]. The UPR, activated by misfolded proteins, initially resolves ER stress [17,18] but becomes pro-apoptotic when prolonged [7], as seen in lung I/R injury where persistent UPR activation contributes to cell death [19]. As the ER's primary sensor of protein homeostasis imbalance, TXNDC5 is uniquely positioned to respond to I/R-induced stress. Therefore, it is highly likely that TXNDC5, activated during the excessive ER response to I/R, plays a significant role in the PEB damage.

To address these knowledge gaps, we investigated TXNDC5′s role in LIRI-induced endothelial dysfunction. Our results demonstrate the following: (1) TXNDC5 impairs vascular integrity *in vitro* and *in vivo*, with its knockdown restoring endothelial function; (2) ATF6 activation during ER stress directly drives TXNDC5 transcription, triggering endothelial apoptosis and barrier disruption; and (3) clinically, TXNDC5 expression levels not only predict postoperative LIRI development but also correlate with disease severity, establishing its potential as a novel diagnostic biomarker.

## Materials and methods

### Plasma protein detection in clinical cohort

Given that LIRI is a clinically relevant complication of cardiopulmonary bypass (CPB) [20,21], we selected this setting to investigate the role of TXNDC5. The study cohort was derived from our PBPMARDS study [16], which enrolled adults undergoing valvular CPB surgery at Union Hospital, Tongji Medical College. The study protocol was approved by the Institutional Ethics Committee (approval number: 20200518), and written informed consent was obtained from all participants prior to inclusion. LIRI cases were defined as ARDS onset ≤72 h post-CPB [22]. Cohort 1 (April-July 2021; n = 20 pairs) underwent proteomic profiling at T1 (CPB termination) and T2 (24 h post-CPB) [16], whereas TXNDC5 levels in Cohorts 2 and 3 (August 2021– March 2023; n = 70 pairs) were measured by ELISA (abx384047, Abbexa) at T1. For detailed methodological specifics (LIRI criteria, inclusion/exclusion, confounders, and study cohort flow diagram), please refer to Supplementary Method S1.

### TXNDC5-based prediction model

Potential clinical predictors for LIRI that have been reported in previous studies were collected by trained investigators (Supplementary Method S2) [16]. The clinical data were preliminarily tested using univariable logistic regression. Then, the clinical variables with *P* < 0.05 were entered into a stepwise logistic regression algorithm (SLR) to screen clinical predictors. Finally, a nomogram consisting of the clinical predictors and TXNDC5 was developed and evaluated via receiver operating characteristic, calibration, and decision-curve analysis. In addition, the proposed nomogram was internally validated using 3-, 5-, and 10-fold cross-validation, and its performance was compared with that of a clinical-predictor-only nomogram via net reclassification improvement (NRI) and integrated discrimination improvement (IDI). For detailed methodological specifics, please refer to Supplementary Method S2.

### Sepsis-ARDS RNA expression data

To compare TXNDC5 expression between I/R and infection-induced lung injury, we downloaded plasma-derived differential gene profiles of a sepsis-related ARDS cohort from the Gene Expression Omnibus (GEO) database (https://www.ncbi.nlm.nih.gov/geo/). Whole-blood transcriptomes (GSE32707; 18 sepsis-ARDS, 30 sepsis-only controls, blood was obtained from patients on the day of admission) were retrieved from GEO and analyzed with limma19; significance: *P* < 0.05, |log_2_(fold change)| >0.263.

### Animal models

Male Sprague–Dawley (SD) rats (300 ± 20 g, Beijing Vital River Laboratory, China) were housed under Specific Pathogen Free (SPF) conditions (12/12-h light/dark cycle, 22–24 °C, 60–65 % humidity). After acclimatization, rats were randomly assigned to experimental groups using a computer-generated random number sequence (SAS software; version 9.4; SAS Institute Inc., Cary, NC, USA). The randomization sequence was concealed until the interventions were assigned. The group sizes for each experimental endpoint were listed in Supplementary Method S3. Throughout the study, investigators responsible for data collection (e.g., histological scoring, TUNEL assay analysis, Western blot quantification) and statistical analysis were blinded to the group allocations. The blinding was maintained until the completion of the final data analysis. All procedures were approved by Tongji Medical College (IACUC #2916), conducted in accordance with our institution's guidelines for the care and use of laboratory animals, and reported according to the ARRIVE guidelines. Rats were anesthetized (sodium pentobarbital, 30 mg/kg i.p.) and mechanically ventilated (8 mL/kg tidal volume, 80 breaths/min). The LIRI model was established via left hilar clamping (60 min ischemia) followed by reperfusion, with sham controls undergoing identical procedures without occlusion. A reperfusion duration of 6 h was utilized for the assessment of LIRI pathology, as this period reliably induces significant pulmonary endothelial barrier dysfunction [16].

### TXNDC5 knockdown and anti-inflammatory intervention *in vivo*

The scrambled shRNA control (ShNC) and TXNDC5-targeting shRNA (Sh*Txndc5*) adenoviruses (AAV) were obtained from Heyuan Biotechnology (Shanghai, China). The targeting sequence for Sh*Txndc5* was CCAGGGTCCTAGAGACTTT. After anesthesia, viruses were delivered via intratracheal injection (insulin needle) and allowed 2 weeks for lung infection (details in Supplementary Method S4). To specifically verify the *in vivo* knockdown efficiency of TXNDC5 in pulmonary endothelial cells, endothelial cells were isolated from rat lung tissue by fluorescence-activated cell sorting (FACS). Lung tissues from ShNC and ShTxndc5 rats were enzymatically digested to generate single-cell suspensions. Dead cells were labeled with SYTOX™ Blue Dead Cell Stain (Thermo Fisher Scientific), and endothelial cells were identified using PE-conjugated anti-rat CD31 (BD Pharmingen™, clone TLD-3A12) and BV650-conjugated anti-rat CD45 (BD OptiBuild™, clone OX-1, RUO) antibodies. Labeled cells were sorted using a BD FACS Aria cell sorter, and the sorted endothelial cells were immediately cultured in Endothelial Cell Growth Medium-2 (EGM-2, CC-3162, Lonza) supplemented with growth factors and 10 % fetal bovine serum (FBS) under standard conditions (37 °C, 5 % CO_2_). Subsequently, TXNDC5 knockdown efficiency was verified by Western blotting (WB) and quantitative PCR (qPCR) analyses.

Dexamethasone (DEX) has been applied in the treatment of ARDS with encouraging results [23,24]. In a rat model, DEX was administered intraperitoneally at a dose of 1 mg/kg 1 h prior to the induction of LIRI [23,24].

### Cell models and interventions targeting TXNDC5, ATF6, and ER stress levels

Human umbilical vein endothelial cells (HUVECs) were purchased from ProCell (Wuhan, China) and cultured in DMEM (11965118, Gibco, USA) supplemented with 10 % FBS and 1 % penicillin/streptomycin at 37 °C with 5 % CO_2_. Primary rat pulmonary microvascular endothelial cells (PMVECs) were isolated from lung tissue using FACS, and primary alveolar epithelial cells (AT1 and AT2) were purchased from ScienceCell Research Laboratories (Catalog No. R3200, USA). The oxygen-glucose deprivation/reoxygenation (OGD/R) model was established by incubating cells in glucose-free medium (11966025, Gibco, USA) under hypoxia (94 % N_2_/1% O_2_) for 6 h, followed by 12 h reoxygenation in complete medium (5 % CO_2_) at 37 ℃.

For mechanistic studies, cells were either (1) infected with AAV-ShTXNDC5(Heyuan Biotechnology, MOI = 1 × 10^5^, 72 h) in the presence of 5 μg/mL polybrene (target sequence: CCAGGGTCCTCGGGACTTT), (2) transfected with pCMV6-Entry-hTXNDC5 using Lipofectamine 3000 for 48 h (Thermo Fisher Scientific), or (3) pre-treated with 5 mM 4-PBA (0.5 h) or 5 μM Ceapin-A7 (4 h), prior to OGD exposure. The vehicle control (DMSO) was validated in preliminary experiments showing no detectable effects on cell viability or morphology compared to untreated controls.

### LC-MS/MS-based proteomic experiment

Lung tissue samples from LIRI and Sham rats were analyzed using LC-MS/MS-based proteomics. Peptides were labeled with tandem mass tag reagents following the manufacturer's protocol, desalted using Sep-Pak C18 columns, and fractionated by high-pH reverse-phase high-performance liquid chromatography. The resulting 15 peptide fractions were vacuum-dried prior to LC-MS/MS analysis. Detailed protocols are provided in Supplementary Method S5.

### Single-cell transcriptome analysis

Single-cell transcriptome profiling was performed using the DNBelab C-TaiM4 platform (BGI, China). Viable single-cell suspensions (>80 % viability) from rat lung tissues were loaded onto microfluidic chips for gel bead-in-emulsion (GEM) generation. The platform's dual-bead capture system enabled efficient mRNA capture and barcoding during reverse transcription. Following GEM breakup and cDNA purification, libraries were constructed and sequenced on BGI platforms (PE100/150 mode, ≥50,000 reads/cell). We employed the Seurat toolkit for clustering analysis after quality control and normalization, defining cell populations with established markers and examining TXNDC5 expression among them.

### Histopathological and ultrastructural analysis

Left lung tissues were collected post-reperfusion and processed for both hematoxylin and eosin (H&E) staining and electron microscopy. For histology, samples were fixed in 4 % paraformaldehyde, embedded in paraffin, and stained with H&E for semiquantitative assessment of inflammatory infiltration, edema, hemorrhage, and alveolar septal thickening [25,26], with inflammatory cells counted in 10 random 400 × fields [27]. For transmission electron microscopy, tissue fragments (1–2 mm^3^) were fixed in glutaraldehyde, post-fixed in osmium tetroxide, and embedded in Epon812. Ultrathin sections (70 nm) were stained with uranyl acetate/lead citrate and imaged using H-600 microscope (Hitachi, Ltd. Tokyo, Japan).

### Pulmonary endothelial permeability assay *in vivo/vitro*

Vascular permeability was evaluated *in vivo* using Evans blue-albumin extravasation, lung wet/dry weight (W/D) ratio, and junctional proteins (VE-cadherin, ZO-1, claudin-5) [28]. Left lungs were weighed fresh and after 56 °C desiccation for W/D ratio calculation. Evans blue dye (20 mg/kg) was intravenously administered post-reperfusion, with concentration determined spectrophotometrically (OD620 − [1.426 × OD740 + 0.03]) [29]. The NO content in lung tissue homogenates and serum was quantified by measuring nitrate (NO_3_^−^) and nitrite (NO_2_^−^) levels using a colorimetric method (Beyotime, China; S0023).

*In vitro*, endothelial barrier function was assessed by transendothelial electrical resistance (TEER) in HUVEC monolayers (6 × 10^4^ cells/mL) cultured in Transwell inserts (200 μL upper/600 μL lower chamber), with values normalized to controls.

### Apoptosis analysis

Apoptosis was assessed by TUNEL staining in lung tissues and flow cytometry in HUVECs. For TUNEL, paraffin sections were stained and imaged by confocal microscopy (Olympus, Tokyo, Japan), with apoptotic index calculated as TUNEL-positive nuclei/total nuclei. For flow cytometry (Beckman Coulter, Inc. California, USA), HUVECs and primary PMVECs were stained with Annexin V-FITC/PI or Annexin V-APC/7-AAD and analyzed for viability (double-negative), early apoptosis (Annexin V^+^&7-AAD^-^/PI^-^), late apoptosis (double-positive), and necrosis (PI^+^/7-AAD^+^&Annexin V-) (Supplementary Method S6). Each apoptosis assay was performed as an independent experiment using separate cell passages and reagent batches.

### Measurement of inflammatory and oxidative stress markers

Myeloperoxidase (MPO) activity was measured to assess neutrophil infiltration using commercial kits (Elabscience, China). Lung homogenate levels of IL-6, IL-10, and ICAM-1 were quantified by ELISA (Neobioscience/ABMART, China). Malondialdehyde (MDA) concentration, an indicator of oxidative stress, was determined using a standardized assay (Elabscience, China). All assays were performed according to the manufacturers' protocols.

### Western blot and immunofluorescence analysis

Western blot and immunofluorescence were performed as described in Supplementary Method S7. Briefly, proteins from lung tissues and HUVECs were extracted, separated by SDS-PAGE, and probed with specific antibodies. For immunofluorescence, lung sections were stained with primary antibodies and fluorescent secondary antibodies corresponding to the species and visualized by confocal microscopy.

### Real-time quantitative Polymerase chain reaction (RT-qPCR)

Transcript levels of *Txndc5* and *Actb* were quantified by RT-qPCR (Bio-Rad, California, USA) using validated primers (NCBI Primer-BLAST). Thermal cycling conditions followed standard protocols, and data were analyzed with IQ5 software (Bio-Rad, Maastricht, The Netherlands). See Supplementary Method S8 for details.

### Co-immunoprecipitation (Co-IP)

Cells pre-treated with AAV-ShTXNDC5 or AAV-ShNC and OGD/R were subjected to Co-IP to assess changes in the binding level of the HSP90/t-eNOS complex. Protein A/G Magnetic Beads (MCE, HY-K0202A) were used according to the manufacturer’s instructions. Antibodies: HSP90 (1:500, Cat#: 13171–1-AP, Proteintech, Illinois, USA), t-eNOS (1:1000, Cat#: A20985, ABclonal, Wuhan, China). The negative control group was treated with IgG to eliminate interference from nonspecific binding.

### Cycloheximide (CHX) chase assay

Cells pre-treated with AAV-ShTxndc5 or AAV-ShNC and OGD/R were analyzed by CHX assay to examine changes in t-eNOS protein stability. Cells were plated in 12-well plates, and after 24 h of adherening, the cells were treated with DMSO or 20 μM CHX for another 0, 0.5, 1, 2, and 4 h, followed by sample collection and Western blot analysis of t-eNOS protein expression.

### Chromatin immunoprecipitation sequencing (ChIP-seq) analysis

Chromatin immunoprecipitation was performed in HUVECs using anti-ATF6 antibody (Abcam, Cambridge, UK) after cross-linking with 1 % formaldehyde and sonication (200–500 bp fragments) [30]. Libraries were prepared (I NEXTFLEX® Kit) and sequenced (Illumina Novaseq 6000, PE 150). The raw ChIP-seq data have been deposited in the Sequence Read Archive under accession number PRJNA1354471. For validation, ChIP-qPCR was conducted using specific primers. Complete methods are described in Supplementary Method S9.

### Statistical analyses

All data were tested for normality using the Shapiro–Wilk test. Data conforming to normal distribution were analyzed using Student's *t*-test (two-group comparisons) or one-way ANOVA with Tukey's post hoc test (multi-group comparisons). Data violating normality assumptions were analyzed using the Wilcoxon rank sum exact test. Multiple comparison corrections were systematically applied where relevant. Data are expressed as mean ± SEM. Methodological details for constructing the TXNDC5-based nomogram model are provided in Supplementary Method S2. Analyses were performed in R software (version 4.2.0; Vienna, Austria). A *P*-value of <0.05 was considered statistically significant, and exact *P*-values for all statistical comparisons are provided.

## Results

### Marked PEB injury induced by LIRI in rats

**Fig. 1:**
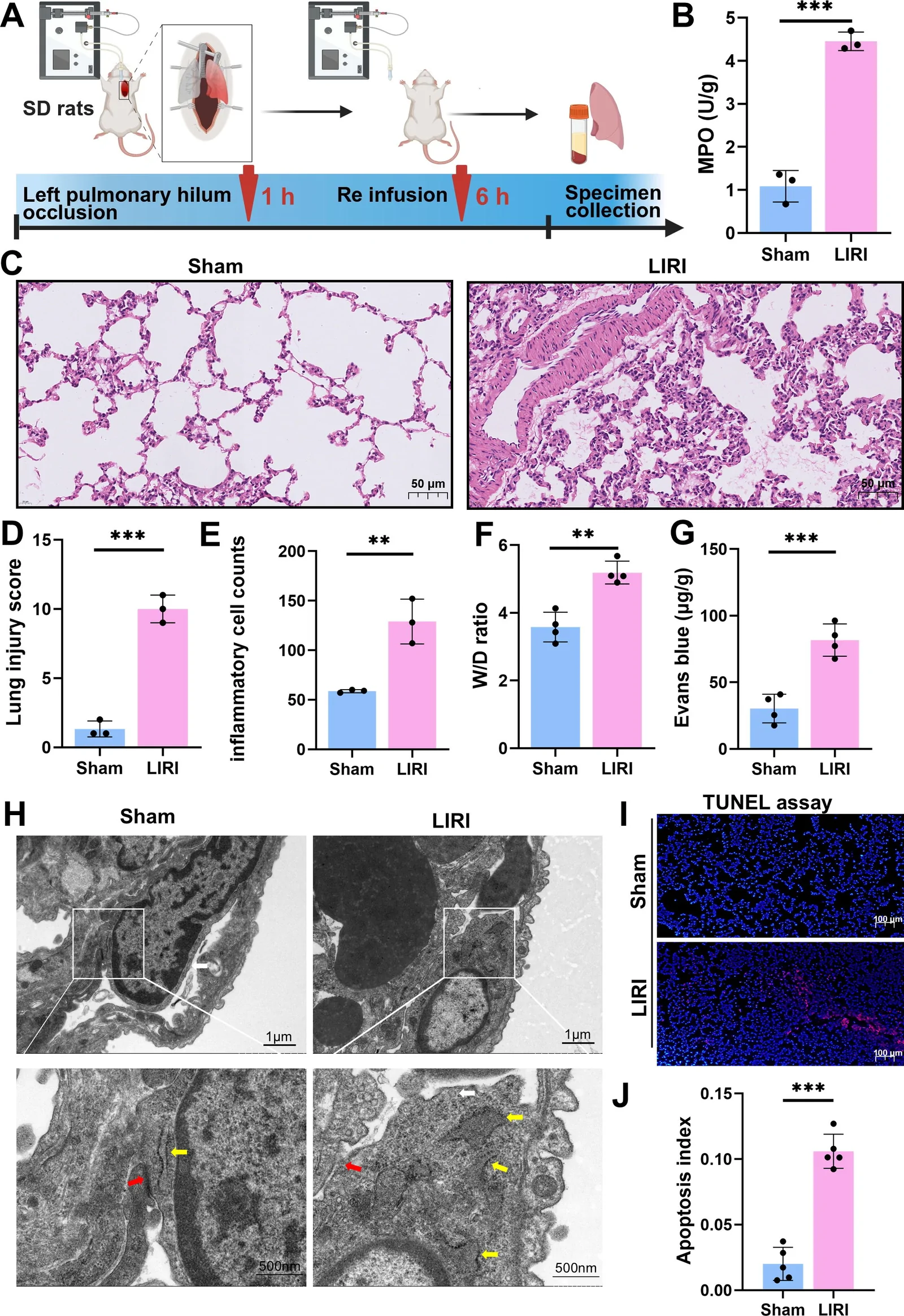
**Marked endothelial barrier injury induced by lung ischemia–reperfusion injury (LIRI) in rats.** (A) Schematic of the experimental LIRI model: left pulmonary artery occlusion for 1 h followed by 6 h reperfusion; Sham-operated rats served as controls. (B) Myeloperoxidase (MPO) activity (U/g) in lung tissue of Sham and LIRI group, indicating neutrophil infiltration (n = 3 per group). (C) Representative hematoxylin-eosin (H&E) stained lung sections showing histological changes in Sham and LIRI group (Scale bar = 50 µm). (D) Semiquantitative lung injury scores from blinded assessment of inflammatory cell infiltration, edema, hemorrhage, and septal thickening (≥6 random 200x fields per rat; n = 3). Infiltration of inflammatory cells, edema, hemorrhage, and alveolar septal thickening were each scored on a 0- to 4-point scale. (E) The amount of inflammatory cells (sum of 10 400x field) in lung tissues from Sham and LIRI group (n = 3 per group). (F) Wet-to-dry (W/D) weight ratio of lungs in Sham and LIRI group (n = 4 per group). (G) Evans blue lung leakage ratio (μg/g wet lung tissue) of lungs in Sham and LIRI group (n = 4 per group). (H) Representative images of transmission electron microscopy examination of lung tissue in Sham and LIRI groups. White arrows: phagocytic vesicles in vascular lumen endothelial cells; Yellow arrows: endoplasmic reticulum structures; Red arrows: tight junction structures of the endothelial barrier. (Scale bar = 1 µm and 500 nm). (I) TUNEL staining in lung tissue cells of Sham and LIRI rats. The positive apoptotic cell nuclei were red in color, and the non-apoptotic cell nuclei were blue in color. (Scale bar = 100 µm). (J) Quantification of TUNEL-positive cells as a percentage of total nuclei in LIRI and Sham group (10.6 % vs 2.8 %, n = 5 per group). Note: data are presented as mean ± SEM. ns: *P* ≥ 0.05; *P < 0.05; **P < 0.01; ***P < 0.001 compared to Sham. All experiments were performed with at least three biological replicates.

The LIRI rat model (Fig. 1A) demonstrated significant PEB injury compared to sham controls. Histological analysis revealed intact alveolar architecture in sham groups’ lungs, whereas LIRI caused pronounced inflammatory infiltration, alveolar disruption, and interstitial congestion (Fig. 1C). Quantitative analysis showed elevated lung injury scores, inflammatory cell counts, and MPO activity in LIRI versus sham groups (Fig. 1B, D, E). Increased W/D ratios and Evans blue leakage confirmed vascular permeability dysfunction (Fig. 1F, G). Ultrastructural analysis revealed endothelial ER dilation (yellow arrows), phagocytic vesicle accumulation, and disrupted tight junctions (red arrows) in LIRI (Fig. 1H). TUNEL staining showed significantly higher apoptosis in LIRI lungs (10.6 % vs. 2.8 %, *P* < 0.001; Fig. 1I, J), confirming extensive PEB injury.

### TXNDC5 was activated in pulmonary endothelial cells after I/R in rats

**Fig. 2:**
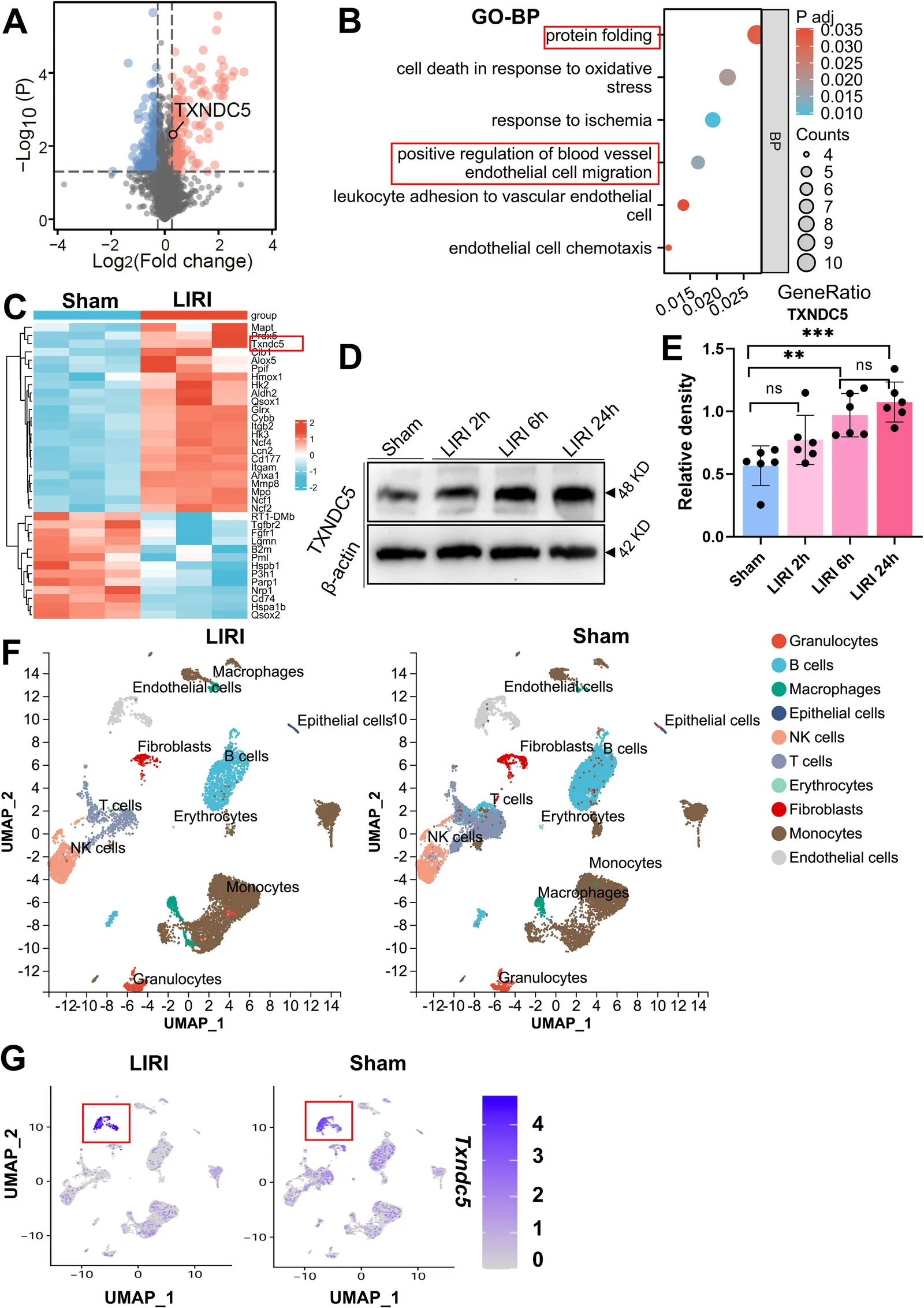
**TXNDC5 is activated in pulmonary endothelial cells after LIRI.** (A) Volcano plot showing differential gene expression between Sham and LIRI groups, with TXNDC5 highlighted (n = 3 per group). (B) Gene Ontology (GO) analysis indicating the biological processes (BP) enriched in the differentially expressed genes, including protein folding, response to ischemia, and endothelial cell migration. (C) Heatmap of differentially expressed genes involved in the enrichment pathway in Sham and LIRI groups, with red indicating upregulation and blue indicating downregulation. (D) Western blot of TXNDC5 protein expression in lung tissue at different time points (2 h, 6 h, 24 h) post-LIRI compared to Sham. β-actin was used as a loading control. (E) Quantification of TXNDC5 relative density showing significant upregulation at 6 h and 24 h post-LIRI (n = 6 per group). (F) UMAP projection of single-cell RNA sequencing data from LIRI and Sham rat lungs, identifying 10 major cell populations (n = 2 per group). (G) UMAP plots showing predominant enrichment of *Txndc5* transcripts specifically in endothelial cells, with minimal expression in other pulmonary cell types. Note: data are presented as mean ± SEM. ns: *P* ≥ 0.05; *P < 0.05; **P < 0.01; ***P < 0.001. All experiments were performed with at least three biological replicates.

Proteomic analysis revealed significant upregulation of TXNDC5 in LIRI lung tissue (Fig. 2A). KEGG analysis identified neutrophil extracellular trap formation, cell adhesion molecules, and apoptosis as most significantly altered (Fig. S1). GO analysis showed enrichment in protein folding and endothelial cell migration pathways (Fig. 2B), with heatmap visualization confirming TXNDC5 overexpression in LIRI (Fig. 2C). Western blot demonstrated increased TXNDC5 protein levels at 6 h and 24 h post-reperfusion (Fig. 2D, E). Therefore, the 6 h time point was selected for subsequent studies to align the analysis of TXNDC5 function with the phase of barrier injury.

Western blot data (Fig. S2) showing TXNDC5 protein was most significantly upregulated in primary rat PMVECs after OGD/R, with minimal changes in primary alveolar epithelial cells (AT1 and AT2). Importantly, in our established OGD/R model using primary rat PMVECs, we confirmed characteristic endothelial dysfunction evidenced by downregulation of HSP90/eNOS expression, decreased TEER, and significantly increased apoptosis (Fig. S3). This cell-type-specific protein expression pattern was further confirmed and extended at the transcriptional level by single-cell RNA sequencing (scRNA-seq) of lungs from I/R-injured rats. Cell populations were identified through Uniform Manifold Approximation and Projection using known marker genes, resolving 10 distinct types including granulocytes, immune cells (B, T, NK, macrophages, and monocytes), erythrocytes, epithelial cells, endothelial cells, and fibroblasts (Fig. 2F). This comprehensive mapping revealed that LIRI significantly perturbs the cellular architecture of the lung, providing a foundation for investigating its impact on specific cell types. Notably, Txndc5 transcripts showed predominant enrichment in endothelial cells, with markedly lower expression across all other pulmonary cell types (Fig. 2G). These combined findings from primary cell models and *in vivo* single-cell transcriptomics robustly identify the pulmonary endothelium as the major site of TXNDC5 activation in LIRI.

### *In vitro* evidence for TXNDC5 regulating endothelial function via HSP90/eNOS stability

**Fig. 3:**
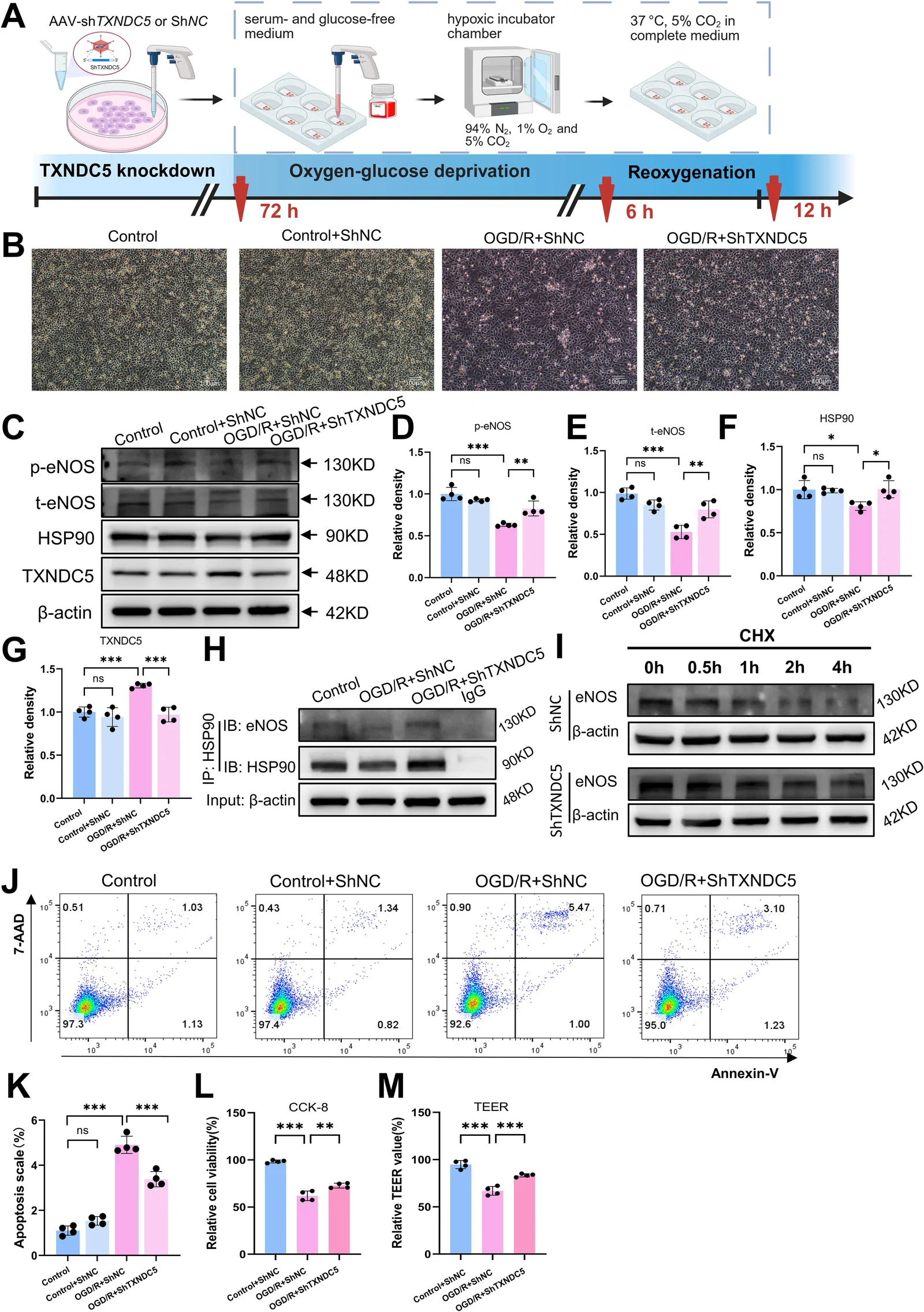
**TXNDC5 regulates endothelial barrier function through HSP90/eNOS complex stability*****in vitro.*** (A) Schematic of the *in vitro* OGD/R model in HUVECs with TXNDC5 knockdown (AAV-ShTXNDC5) or control (ShNC) treatment. (B) Representative phase-contrast images of HUVECs in control, control + ShNC, OGD/R + ShNC, and OGD/R + ShTXNDC5 groups (Scale bar = 100 µm). (C) Western blot analysis of HSP90, phosphorylated eNOS (Ser^1177^), total eNOS, and TXNDC5 protein expression in control, control + ShNC, OGD/R + ShNC, and OGD/R + ShTXNDC5 groups, with β-actin as a loading control. (D-G) Quantification of relative protein levels of p-eNOS (D), t-eNOS (E), HSP90(F) and TXNDC5 (G), respectively (n = 4 per group). (H) Co-immunoprecipitation demonstrate enhanced interaction between HSP90 and t-eNOS upon TXNDC5 knockdown. (I) Cycloheximide (CHX) chase assay revealing that TXNDC5 knockdown prolongs eNOS protein half-life. (J) Flow cytometry of apoptosis using Annexin V/7-AAD staining; double-positive cells indicate late apoptosis. (K) Statistical plot of the proportion of late apoptotic cells in control, control + ShNC, OGD/R + ShNC, and OGD/R + ShTXNDC5 groups (n = 4 per group). (L) Cell viability measured by CCK-8 assay and expressed as percentage of control (n = 4 per group). (M) Endothelial barrier function assessed by transendothelial electrical resistance (TEER) and expressed as percentage of baseline (n = 4 per group). Note: data are presented as mean ± SEM. ns: *P* ≥ 0.05; *P < 0.05; **P < 0.01; ***P < 0.001. All experiments were performed with at least three biological replicates.

To investigate the role of TXNDC5 in LIRI, we established an I/R injury model in HUVECs by OGD/R and utilized AAV-ShTXNDC5 to knock down TXNDC5 expression (Fig. 3A). Western blot and PCR analysis demonstrated that TXNDC5 was knocked down (Fig. S4) and significantly attenuated cellular injury (Fig. 3B). Given the established role of eNOS/HSP90 in endothelial protection [14,31], we examined their regulation by TXNDC5, which is also highly expressed in endothelial cells. Mechanistically, TXNDC5 silencing restored HSP90, p-eNOS (Ser^1177^), and t-eNOS expression (Western blot; Fig. 3C-G) and promoted the interaction between HSP90 and t-eNOS (Co-IP; Fig. 3H). In contrast, TXNDC5 knockdown showed minimal effect on p-eNOS (Thr^495^), which remained stable following injury (Fig. S5). Furthermore, CHX chase experiments demonstrated that knockdown of TXNDC5 enhances the stability of t-eNOS protein (Fig. 3I). Knockdown of TXNDC5 also reduced apoptosis (flow cytometry with Annexin V/7AAD staining; Fig. 3J, K) and improved cell viability (CCK-8 assay; Fig. 3L) and barrier function (TEER assay; Fig. 3M) compared to ShNC groups.

### TXNDC5 knockdown alleviates LIRI *in vivo* via the HSP90/eNOS complex

**Fig. 4:**
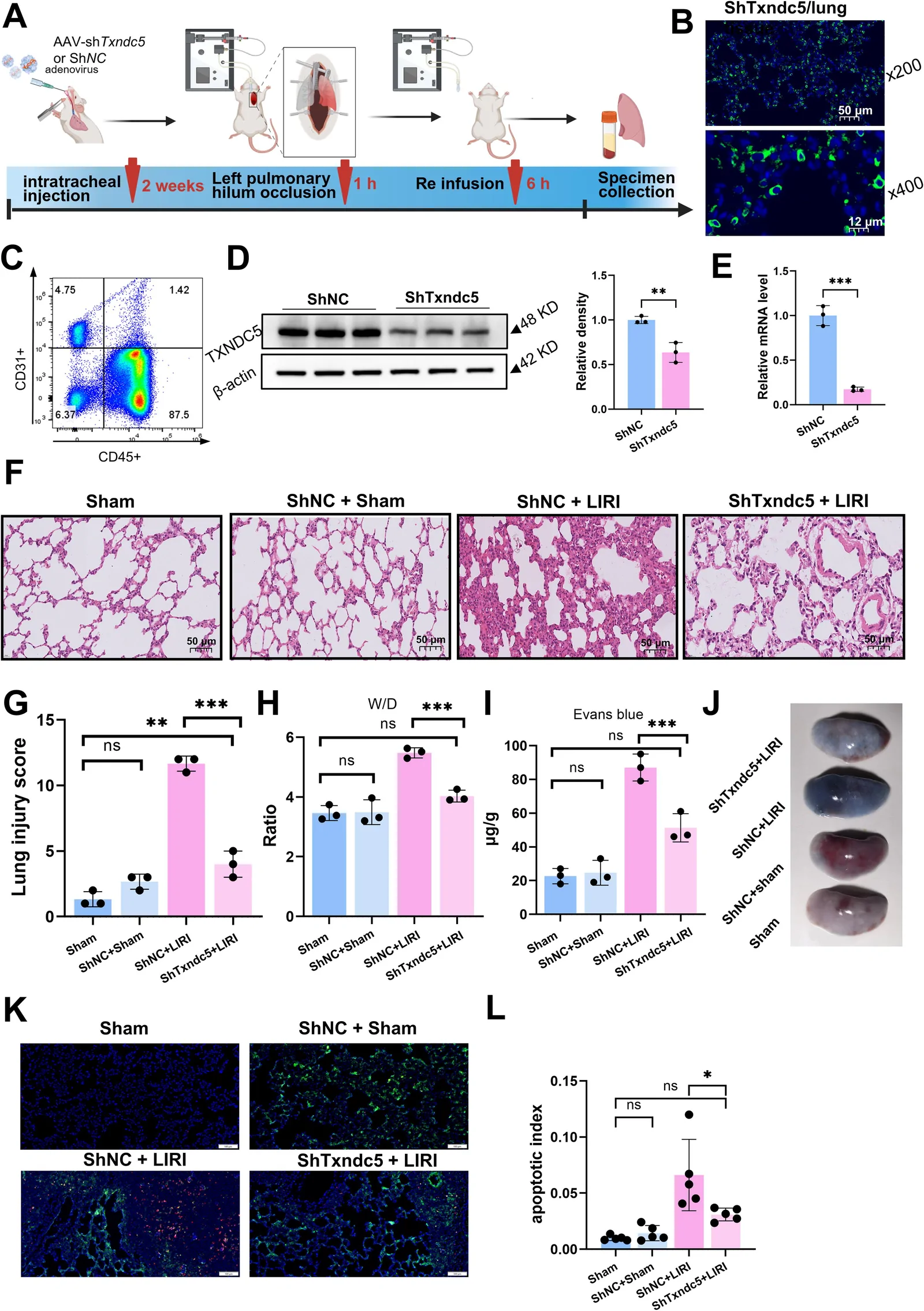
**TXNDC5 knockdown alleviates lung injury in LIRI rats.** (A) Experimental timeline showing intratracheal delivery of ShNC or ShTxndc5 for 2 weeks, followed by left pulmonary artery occlusion (1 h) and reperfusion (6 h). (B) Frozen sections of rat lung tissues 2 weeks after intratracheal injection of AAV, showing scattered green fluorescence indicating successful viral delivery and lung infection. (C) Representative flow cytometry gating strategy used to isolate pulmonary endothelial cells from rat lung tissue. Single-cell suspensions were stained with PE-conjugated anti-rat CD31 and BV650-conjugated anti-rat CD45 antibodies. Endothelial cells were identified as CD31^+^/CD45^−^ and collected for downstream analyses. (D, E) TXNDC5 knockdown efficiency confirmed by (D) Western blot and (E) qPCR in purified endothelial cells (n = 3 per group). (F) Representative hematoxylin-eosin staining (Scale bar = 50 µm) of lung tissues from indicated groups. (G) Lung injury scores from blinded histopathological evaluation of at least six random 200x fields per lung, based on inflammatory cell infiltration, edema, hemorrhage, and alveolar septal thickening (each scored 0–4; n = 3 per group). (H) Lung wet/dry (W/D) ratio following I/R and TXNDC5 knockdown (n = 3 per group). (I) Evans blue lung leakage ratio (μg/g wet lung tissue) following I/R injury and TXNDC5 downregulation (n = 3 per group). (J) Representative images of lung sections stained with Evans blue in the Sham, ShNC + Sham, ShNC + LIRI, ShTxndc5 + LIRI groups. (K-L) TUNEL staining of lung tissues (scale bar = 100 µm) and quantification of apoptotic index based on TUNEL‑positive cells in Sham, ShNC + Sham, ShNC + LIRI, ShTxndc5 + LIRI (1.0 % vs 1.5 % vs 6.7 % vs 3.1 %, n = 5 per group). Note: data are presented as mean ± SEM. ns: *P* ≥ 0.05; *P < 0.05; **P < 0.01; ***P < 0.001. All experiments were performed with at least three biological replicates.

For *in vivo* assessment, SD rats were randomly allocated to receive tracheal injections of either a control virus (ShNC) or a targeting virus (ShTxndc5) (Fig. 4A). Following a 2-week period of viral infection, fluorescence microscopy of lung sections confirmed widespread AAV transduction (Fig. 4B). Knockdown efficiency was further confirmed both in whole lung homogenates and, more specifically, in FACS-purified CD31^+^/CD45^−^ pulmonary endothelial cells (Fig. 4C). qPCR and Western blot analysis in whole lung homogenates (Fig. S6) and purified pulmonary endothelial cells (Fig. 4D-E), verifying successful endothelial targeting.

Control virus (ShNC) administration showed no significant effects on lung injury parameters compared to sham (*P* > 0.05, Fig. 4F-I), confirming the safety of AAV delivery. In contrast, TXNDC5 knockdown (ShTxndc5 + LIRI) significantly attenuated LIRI-induced pathology, demonstrating (1) reduced lung injury scores (Fig. 4F, G) and inflammatory cell counts (Fig. S7) and (2) improved PEB function (W/D ratio and Evans blue level, Fig. 4H-J) and apoptosis (TUNEL assay, Fig. 4K, L) vs. ShNC + LIRI. These results establish that TXNDC5 silencing protects against LIRI-induced lung injury.

**Fig. 5:**
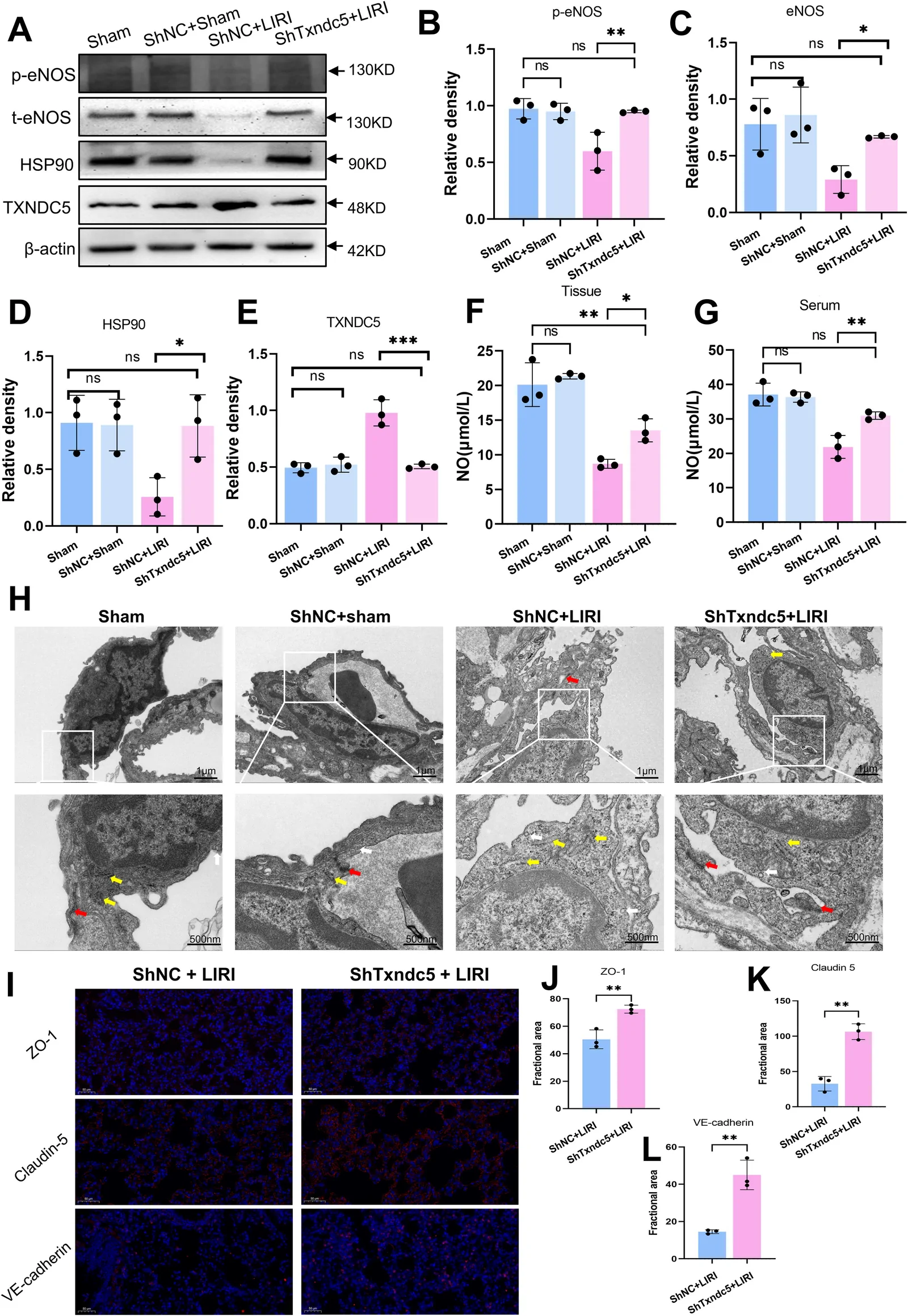
**TXNDC5 knockdown alleviates LIRI by restoring HSP90/eNOS complex*****in vivo.*** (A) Western blot analysis of TXNDC5, HSP90, p-eNOS (Ser^1177^), and t-eNOS in lung tissues after ischemia–reperfusion injury and TXNDC5 knockdown. (B-E) Quantification of (B) p-eNOS, (C) t-eNOS, (D) HSP90, and (E) TXNDC5 protein expression in Sham, ShNC + Sham, ShNC + LIRI, ShTxndc5 + LIRI groups (n = 3 per group). (F, G) NO levels in (F) lung tissue homogenates and (G) serum, decreased after LIRI, were recovered by TXNDC5 knockdown. (H) Representative transmission electron microscopy images of lung tissues. White arrows: phagocytic vesicles in endothelial cells; Yellow arrows: endoplasmic reticulum; Red arrows: endothelial tight junctions (Scale bars = 1 µm and 500 nm). (I-L) Immunofluorescence analysis (I) demonstrated that TXNDC5 knockdown preserved the continuity (I) and fractional area (J-L) of junctional proteins (ZO-1, claudin-5, and VE-cadherin) (Scale bars = 50 µm) (n = 3 per group). Note: data are presented as mean ± SEM. ns: *P* ≥ 0.05; *P < 0.05; **P < 0.01; ***P < 0.001. All experiments were performed with at least three biological replicates.

Western blot analysis showed a significant reduction of TXNDC5 expression in the ShTxndc5 + LIRI group compared to the ShNC + LIRI group (Fig. 5A, E). The expression levels of the protective proteins p-eNOS (Ser^1177^), t-eNOS, and HSP90 were decreased in the ShNC + LIRI group but were restored in the ShTxndc5 + LIRI group, indicating that TXNDC5 knockdown stabilizes the expression of eNOS and HSP90 (Fig. 5B-D). Under I/R injury conditions, dysfunctional eNOS results in insufficient NO production, which fails to maintain vasodilation and barrier protection [32,33]. NO expression in lung tissue homogenates and serum was decreased in the ShNC + LIRI group but restored in the ShTxndc5 + LIRI group (Fig. 5F, G). Transmission electron microscopy examination (Fig. 5H) revealed that the ShNC + LIRI group had disrupted tight junctions and increased swelling of the ER, which were ameliorated in the ShTxndc5 + LIRI group. Immunofluorescence analysis revealed that ShTxndc5 treatment significantly mitigated the disruption of the continuity and membrane localization of VE-cadherin, ZO-1, and claudin-5, restoring the fractional area of all three junctional proteins (Fig. 5I-L).

### TXNDC5 suppression inhibits NF-κB signaling and synergizes with dexamethasone to protect against LIRI

**Fig. 6:**
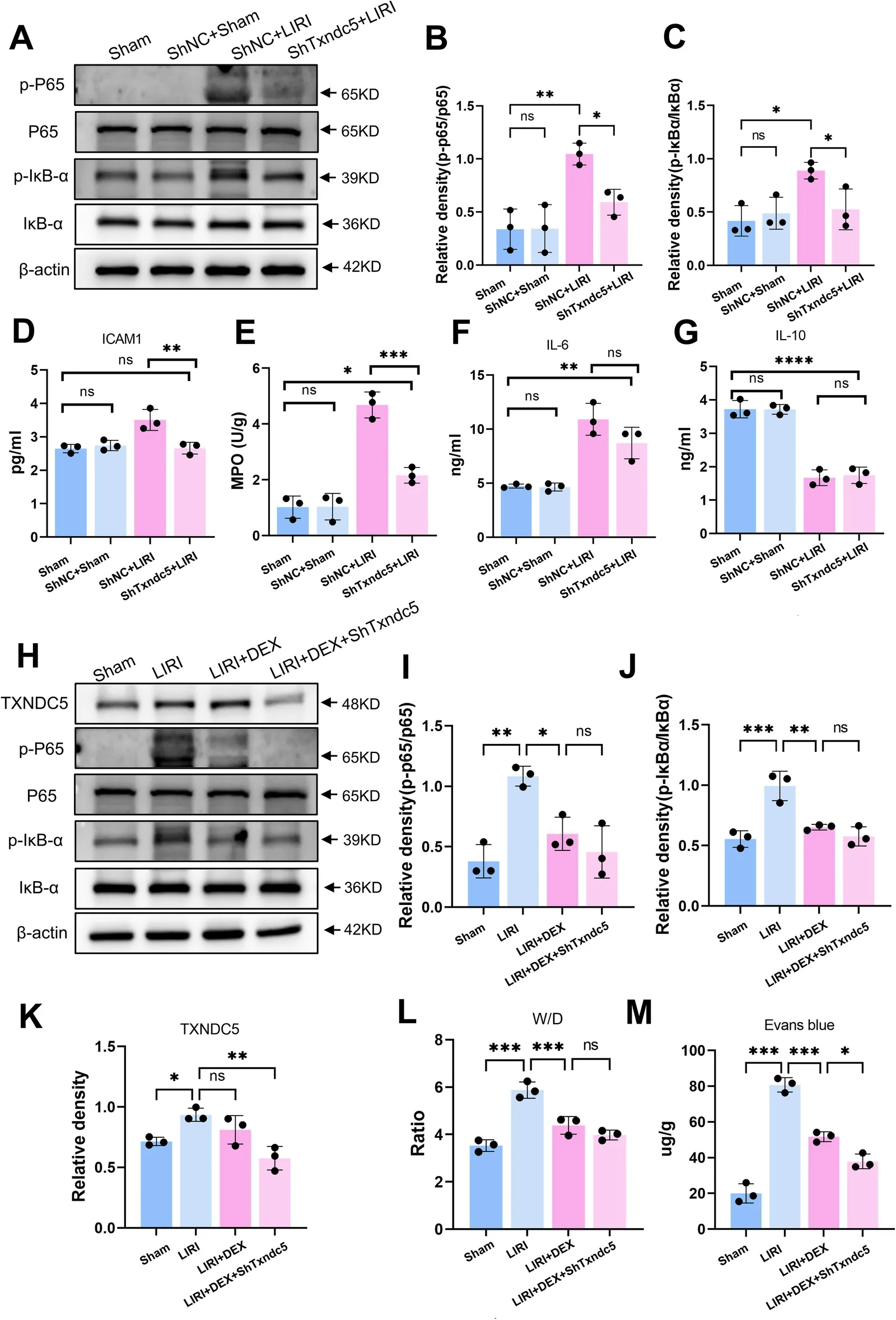
**Combined TXNDC5 knockdown and dexamethasone treatment synergistically protects against LIRI.** (A) Western blot analysis of p-IκBα and p-NF-κB p65 in lung tissues, showing that TXNDC5 knockdown attenuated their LIRI-induced phosphorylation. (B, C) Quantification of p-NF-κB p65 (B) and p-IκBα(C) in Sham, ShNC + Sham, ShNC + LIRI, ShTxndc5 + LIRI groups (n = 3 per group). (D) Quantification of ICAM-1 levels in lung homogenates (n = 3 per group). (E) Myeloperoxidase (MPO) activity (U/g) in lung homogenates (n = 3 per group). (F, G) Quantification of (F) IL-6 and (G) IL-10 levels in lung homogenates (n = 3 per group). (H) Western blot analysis of TXNDC5, p-IκBα, and p-NF-κB p65 in lung tissues from rats subjected to LIRI and treated with Dexamethasone (DEX) or the combination of ShTxndc5 and DEX. (I-K) Quantification of p-NF-κB p65 (I), p-IκBα (J), and TXNDC5(K) levels across treatment groups (n = 3 per group). (L, M) Combined TXNDC5 knockdown and DEX treatment synergistically improved endothelial barrier function, as measured by (L) Lung wet/dry (W/D) ratio and (M) Evans blue leakage assay (n = 3 per group). Note: data are presented as mean ± SEM. ns: *P* ≥ 0.05; *P < 0.05; **P < 0.01; ***P < 0.001. All experiments were performed with at least three biological replicates.

Our investigation into the inflammatory mechanisms of LIRI revealed that TXNDC5 may act as an upstream activator of the NF-κB pathway. Western blot analysis demonstrated that LIRI significantly induced the phosphorylation of IκBα and p65, events that were markedly attenuated by TXNDC5 knockdown (Fig. 6A-C). Consistent with this role in NF-κB, TXNDC5 knockdown significantly mitigated the downstream consequences of this pathway, namely the upregulation of the adhesion molecule ICAM-1 (Fig. 6D) and the oxidative stress marker MDA (Fig. S8), leading to a substantial reduction in neutrophil infiltration (MPO activity) (Fig. 6E). In contrast, levels of the cytokines IL-6 and IL-10 remained unchanged (Fig. 6F, G), indicating that TXNDC5 promotes inflammation primarily through leukocyte adhesion and oxidative damage rather than cytokine signaling.

We further explored the interaction between this pathway and conventional anti-inflammatory therapy. Treatment with DEX effectively suppressed the NF-κB activation (Fig. 6H-J) in LIRI. However, DEX did not alter TXNDC5 expression itself, suggesting its action lies downstream of TXNDC5 activation (Fig. 6K). Strikingly, the combination of DEX and ShTxndc5 resulted in a synergistic protective effect, yielding significantly greater improvement in barrier function (Fig. 6L, M).

### Attenuation of ER stress reduces TXNDC5 levels and ameliorates LIRI

**Fig. 7:**
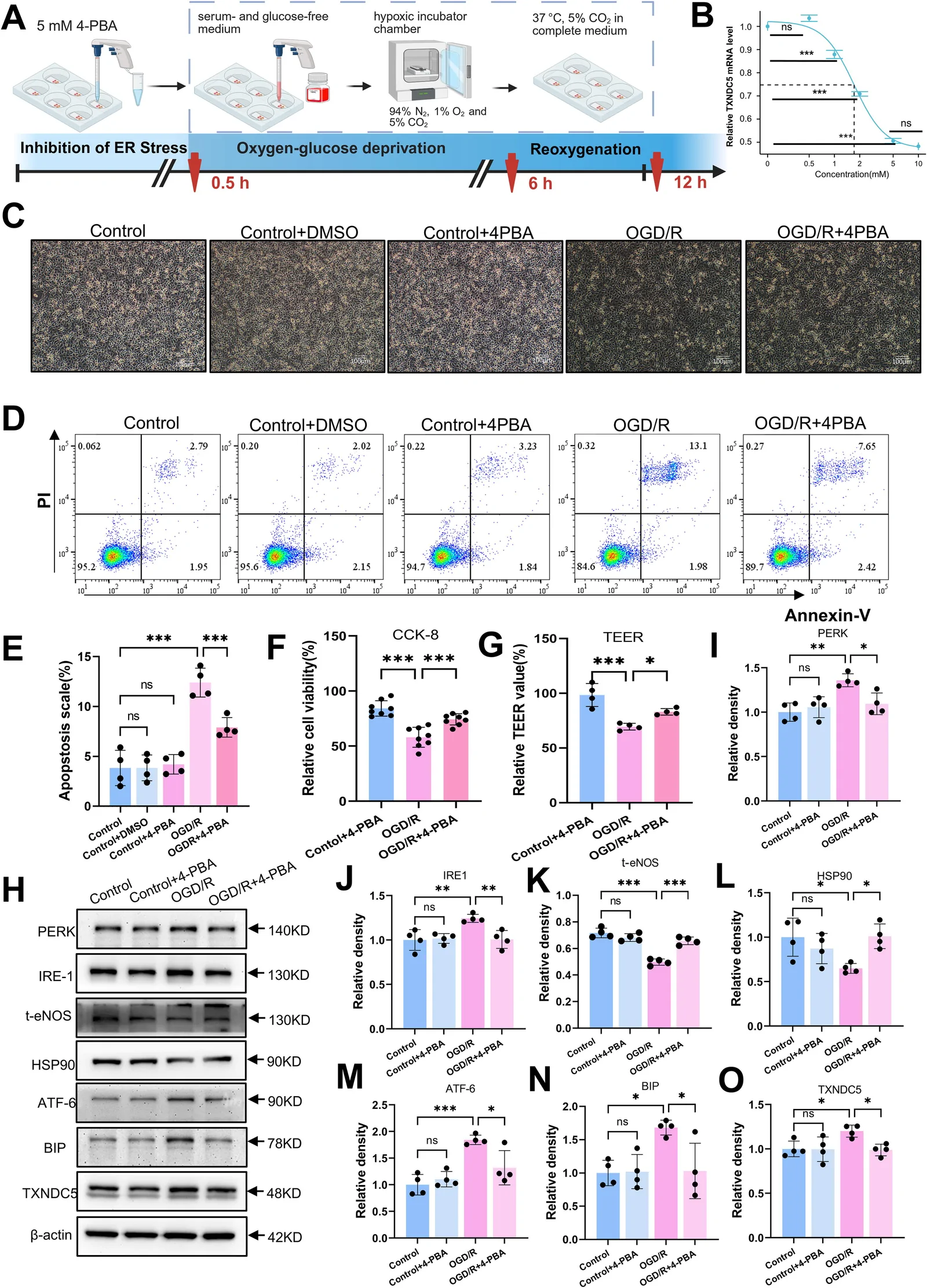
**Activation of endoplasmic reticulum signaling can lead to apoptosis and damage of HUVECs through the TXNDC5-HSP90/eNOS axis.** (A) Experimental timeline of HUVECs pretreatment with 5 mM 4-PBA (or vehicle) for 0.5 h, followed by oxygen-glucose deprivation (OGD) for 6 h and reoxygenation for 12 h (OGD/R). (B) 4-PBA dose-dependently reduced TXNDC5 mRNA levels, with maximal inhibition at 5 mM (n = 3 per group). (C) Representative phase-contrast images of HUVECs in control, control + DMSO, control + 4PBA, OGD/R, and OGD/R + 4PBA groups (Scale bar = 100 µm). (D) Flow cytometry detection of HUVECs apoptosis in control, control + DMSO, control + 4-PBA, OGD/R, OGDR + 4-PBA group. Late apoptotic cells are defined as Annexin-V and PI double-positive. (E) Statistical plot of the proportion of late apoptotic cells in all groups (n = 4 per group). The control + 4-PBA group served as the pharmacological control in subsequent experiments, based on our preliminary validation that the vehicle (DMSO) alone produced no detectable effects (Fig. 7C-E). (F) Cell viability assessed by CCK-8 assay, expressed as percentage of control (n = 8 per group). (G) Endothelial barrier function assessed by relative transendothelial electrical resistance (TEER), expressed as percentage of control (n = 4 per group). (H) Endoplasmic reticulum stress indicators, HSP90/t-eNOS, and TXNDC5 expression were analyzed by western blotting using β-actin as a standardized reference. (I-O) The intensity of (I) PERK, (J) IRE1, (K) t-eNOS, (L) HSP90, (M) ATF6, (N) BIP, and (O) TXNDC5 in four groups was quantified by densitometry with ImageJ software (n = 4 per group). Note: data are presented as mean ± SEM. ns: *P* ≥ 0.05; *P < 0.05; **P < 0.01; ***P < 0.001. All experiments were performed with at least three biological replicates.

Although ER stress activation has been shown to upregulate TXNDC5 in pulmonary fibrosis [34], its role in I/R injury remains unclear. Using the ER stress inhibitor 4-PBA in an OGD/R model (Fig. 7A), we observed that 4-PBA treatment significantly and dose-dependently reduced TXNDC5 transcription (Fig. 7B) while concurrently attenuating OGD/R-induced cell death (Fig. 7C) and apoptosis (flow cytometry; Fig. 7D, E), improving cell viability (CCK-8 assay; Fig. 7F) and barrier function (TEER measurement; Fig. 7G). We used the Control + 4-PBA group as the pharmacological control, having first validated that the vehicle (DMSO) had no detectable effects on cellular states, including viability and apoptosis (Fig. 7C-E). Western blot analysis showed 4-PBA suppressed activation of ER stress markers (BIP, ATF6, IRE1, PERK; Fig. 7H-J, M, N), downregulated TXNDC5 expression (Fig. 7O), and restored HSP90/t-eNOS levels (Fig. 7K, L). These findings demonstrate that pharmacologic ER stress inhibition attenuates LIRI by modulating the TXNDC5-HSP90/eNOS axis.

### Inhibition of the ATF6 pathway ameliorates TXNDC5-driven PEB damage

**Fig. 8:**
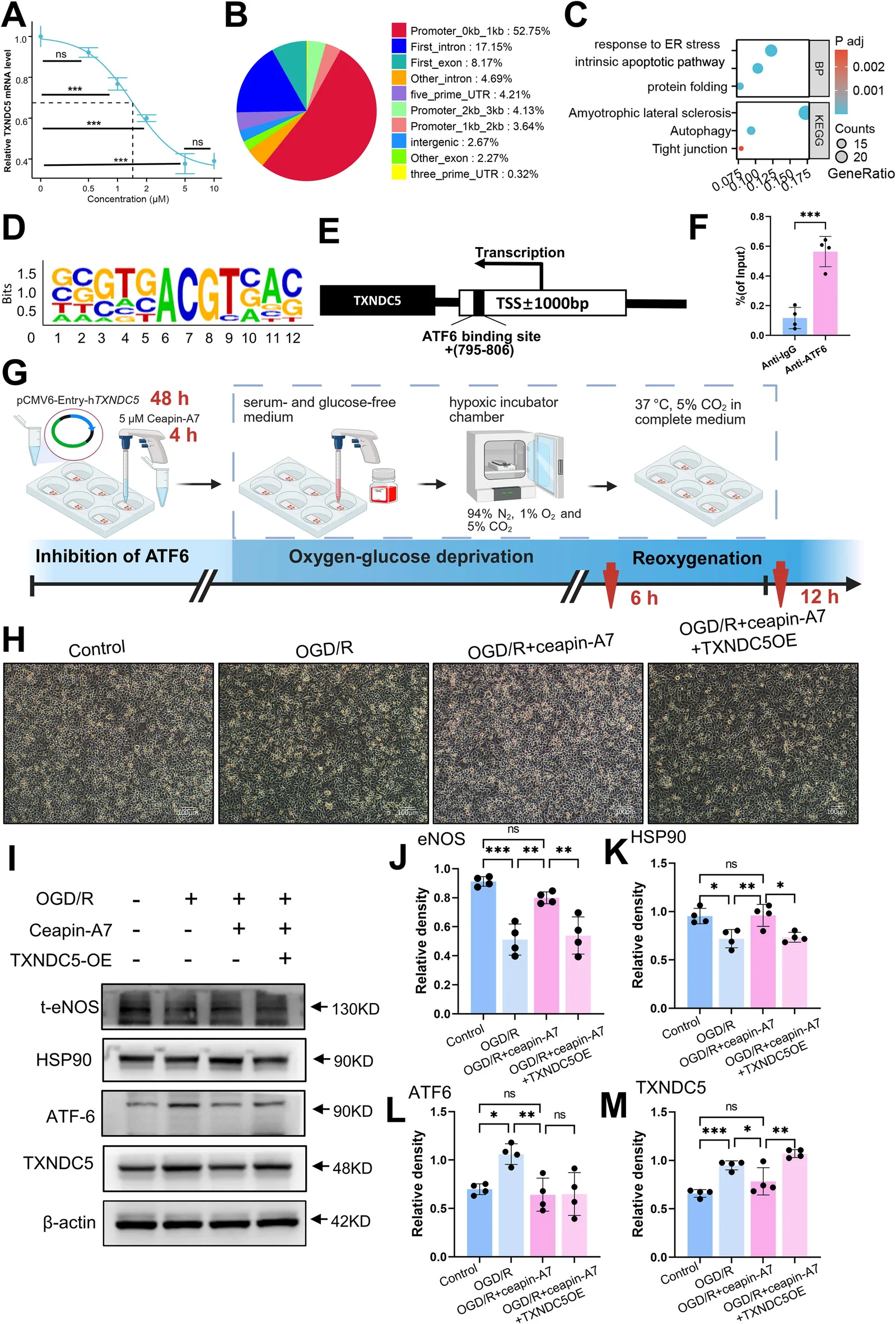
**ATF6-dependent ER stress induces TXNDC5-mediated apoptosis and damage in HUVECs.** (A) Ceapin-A7 dose-dependently reduced TXNDC5 transcription, with maximal inhibition at 5 μM (n = 3 per group). (B) Genomic distribution of ATF6-binding sites in HUVECs following OGD/R modeling, based on annotated genomic regions. (C) GO and KEGG enriched pathways of ATF6-bound genes in HUVECs, including protein folding, response to ER stress, and tight junction. (D) The top de novo motif predicted for ATF6 binding by HOMER analysis. (E) Predicted ATF6-binding site in the TXNDC5 promoter, located at + 795 to + 806 bp relative to the transcription start site (TSS). (F) The interaction between ATF6 and the predicted binding site in the TXNDC5 promoter region assessed by ChIP-qPCR. Compared with the IgG control, the Anti-ATF6 group showed higher enrichment at the target region (n = 4 per group). (G) Experimental timeline: Cells were pretreated with 5 μM Ceapin-A7 (ATF6 inhibitor, 4h) and transfected with TXNDC5 overexpression plasmid (pCMV6-Entry-hTXNDC5, 48 h) prior to OGD (6 h)/reoxygenation (12 h) treatment. (H) Representative phase-contrast images of HUVECs in control, OGD/R, OGD/R + Ceapin-A7 and OGD/R + Ceapin-A7 + TXNDC5-OE (overexpress) groups (Scale bar = 100 µm). Based on our preliminary validation, the vehicle (DMSO) alone produced no detectable effects (Fig. 7C-E). (I) Protein levels of t-eNOS, HSP90, ATF6, and TXNDC5 were assessed by Western blot with β-actin loading controls across four experimental groups. (J-M) The intensity of eNOS (J), HSP90 (K), ATF6 (L), TXNDC5 (M) in four groups was quantified by densitometry with ImageJ software (n = 4 per group). Note: data are presented as mean ± SEM. ns: *P* ≥ 0.05; *P < 0.05; **P < 0.01; ***P < 0.001. All experiments were performed with at least three biological replicates.

Transcription of TXNDC5 exhibited a dose-dependent decrease upon ATF6 inhibition in the OGD/R model (Fig. 8A). Although the ATF6 branch of ER stress signaling regulates TXNDC5 expression in both pulmonary fibrosis [34] and cardiac fibroblast cells [12], its role in endothelial cells remained unknown. Therefore, we performed ChIP-seq analysis of ATF6 in HUVECs subjected to OGD/R conditions. ChIP-seq results revealed that ATF6 was predominantly enriched at the promoter and first intron regions of the genes (Fig. 8B), suggesting its involvement in transcriptional regulation. KEGG pathway analysis revealed that these ATF6-regulated genes were primarily associated with ER protein processing (Fig. 8C), consistent with TXNDC5′s function. ChIP-seq analysis demonstrated a pronounced enrichment peak of ATF6 at the proximal promoter region of TXNDC5 (Fig. S9), indicating potential transcriptional regulation by ATF6. To further validate the regulatory role of ATF6 in TXNDC5 transcription, we used the top-scoring motif predicted by HOMER and aligned it with the ± 1 kb region flanking the transcription start site (TSS) of TXNDC5. This analysis identified a potential ATF6-binding site located at positions + 795 to + 806 relative to the TSS (Fig. 8D, E). To validate the ChIP-seq results, we performed ChIP-qPCR targeting the region near the TXNDC5 TSS, and confirmed the specific binding of ATF6 to this region (Fig. 8F).

Functional studies using Ceapin-A7 (5 μM ATF6 inhibitor, 4 h pretreatment, Fig. 8G) demonstrated that ATF6 inhibition attenuated OGD/R-induced cell death (Fig. 8H), whereas TXNDC5 overexpression reversed this protection. Western blot analysis confirmed Ceapin-A7 reduced ATF6 and TXNDC5 expression while restoring HSP90/eNOS levels (Fig. 8I) and VE-cadherin/ZO-1 (Fig. S10) that were downregulated by OGD/R. However, excessive overexpression of TXNDC5 reversed these protective effects (Fig. 8J-M, S10). These findings establish that ER stress promotes TXNDC5 expression through ATF6 signaling, contributing to endothelial barrier dysfunction in LIRI.

### Plasma TXNDC5 concentration was elevated in patients with LIRI

**Fig. 9:**
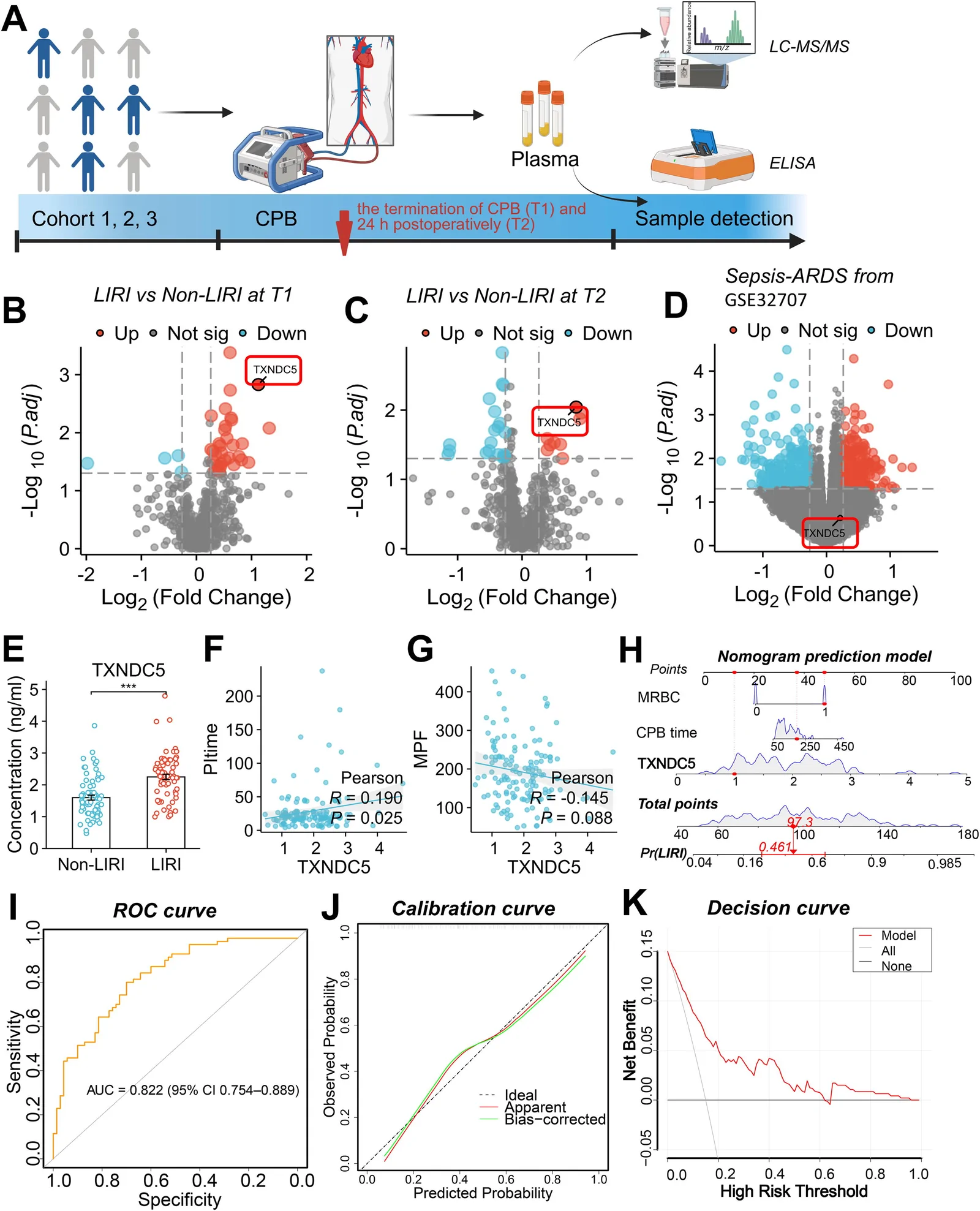
**Identification and validation of TXNDC5 as a predited biomarker for LIRI.** (A) Study design of three cohorts (Cohort 1, 2, and 3) undergoing elective cardiopulmonary bypass (CPB). Plasma was collected at CPB termination (T1) and 24 h post-operation (T2). (B, C) Volcano plots from DIA-proteomic re-analysis of cohort 1 (20 case-control pairs) showing TXNDC5 as a significantly upregulated protein in LIRI patients at (B) T1 and (C) T2. TXNDC5 is highlighted in red. (D) Analysis of the public GSE32707 dataset (18 sepsis-ARDS vs. 30 sepsis-only controls) revealed no significant upregulation of TXNDC5 in sepsis-ARDS. (E) ELISA validation of TXNDC5 in LIRI patients compared to Non-LIRI patients at T1 in patients of cohort 2 and 3. (F, G) Correlation between plasma TXNDC5 levels and (F) intubation time (R = 0.190, p = 0.025) or (G) minimum PaO_2_/FiO_2_ (PF) ratio (R = -0.145, p = 0.088). (H) Nomogram for predicting LIRI probability, incorporating TXNDC5 levels and clinical parameters (massive transfusion of red blood cells [MRBC] and CPB time). The blue waves represent data distribution for each variable. Draw a vertical line from the corresponding axis of each factor to the points axis to acquire the point of this factor. Make a summation of the points for each factor to yield a total score, and the probability of LIRI could be estimated by projecting the total score to the lower probability axis. (I) Receiver operating characteristic (ROC) curve of the TXNDC5-based prediction model (AUC = 0.822, 95 % CI: 0.754–0.889). (J) Calibration plot of the prediction model with a 1000 repetition bootstrap. (K) Decision curve showed the net benefit for the TXNDC5-based prediction model.

A sequence homology comparison (NCBI BLAST) between the human TXNDC5 and the rat TXNDC5 protein was conducted and confirmed high conservation between human and rat TXNDC5 (90 % identity, E≈0; Fig. S11). To characterize LIRI evolution at the protein level, we performed DIA-proteomic sequencing on plasma from patients undergoing CPB. The study included three cohorts undergoing CPB surgery, with samples collected at CPB termination (T1) and 24 h post-operation (T2) (Fig. 9A). Re-analysis of proteomic profiling of Cohort 1 revealed significant TXNDC5 upregulation in patients with LIRI at T1 (*P* < 0.05, |log_2_(FC)|>0.263; Fig. 9B), and T2 (Fig. 9C). A heatmap of protein expression at T1 and T2 was displayed in Fig. S12. To investigate the specificity of TXNDC5 elevation for LIRI, we analyzed the whole-blood RNA expression profiles of patients with sepsis-ARDS and sepsis-only (GSE32707). Notably, TXNDC5 expression was not significantly altered in septic ARDS, suggesting a potentially distinct role in sterile inflammation-driven LIRI (Fig. 9D).

ELISA validation in Cohorts 2–3 confirmed higher TXNDC5 levels in patients with LIRI at T1 (Fig. 9E). TXNDC5 levels correlated positively with intubation time (R = 0.190, *P* = 0.025; Fig. 9F) and negatively with PaO_2_/FiO_2_ (PF) ratio (R = -0.145, *P* = 0.088; Fig. 9G). To assess the predictive utility of TXNDC5, we developed a predictive model incorporating three factors: TXNDC5 protein concentration, massive red blood cell transfusion, and duration of CPB (Fig. 9H). Initial screening of clinical confounders and subsequent SLR selection are detailed in Table S1 and S2. This model achieved an area under the curve (AUC) of 0.822 (95 % CI: 0.754–0.889) (Fig. 9I), with good calibration and net benefit (Fig. 9J, K). Cross-validation (3-, 5-, and 10-fold) for the internal validation consistently yielded AUCs > 0.80 (Table S3). Adding TXNDC5 to the clinical predictor-only model significantly improved reclassification (NRI: 0.457, 95 % CI: 0.266–0.648; IDI: 0.114, 95 % CI 0.060–0.168; both *P* < 0.05) (Table S3).

## Discussion

Our study identifies TXNDC5 as a key regulator of PEB dysfunction in LIRI, implicating an ATF6-mediated ER stress pathway and subsequent disruption of eNOS/HSP90 signaling. By integrating single-cell transcriptome, proteomics profiling, genetic knockdown, and clinical validation, we demonstrate that TXNDC5 not only serves as a mechanistic linchpin in LIRI pathogenesis but also holds promise as a translational therapeutic target.

### TXNDC5 as a novel mediator of LIRI-PEB injury

I/R injury triggers a cascade of oxidative stress, inflammation, and endothelial dysfunction, culminating in tissue damage [35]. The maintenance of PEB integrity is crucial for preserving lung function, as its disruption can lead to increased vascular permeability and pulmonary edema, hallmarks of ALI/ARDS [36,37]. Here, we identify TXNDC5 as a pivotal mediator of PEB disruption in LIRI. First, we observed significant upregulation of TXNDC5 following I/R injury, consistent with previous reports demonstrating its involvement in hypoxia-induced vascular injury [14,38]. Second, mechanistic studies revealed that TXNDC5 selectively impairs endothelial integrity through destabilization of the eNOS/HSP90 complex, distinguishing its function from other PDIs that primarily stabilize protein folding structure. This specificity of disrupting protein stability is further supported by recent work showing TXNDC5-mediated eNOS destabilization in atherosclerosis [14] and vascular calcification [15]. HSP90 serves as a crucial molecular chaperone for eNOS stabilization in endothelium [39], whereas TXNDC5 is reported to influence HSP90 transcription via its PDI activity, consequently modulating eNOS and PEB integrity [14,40]. Third, and most importantly, AAV-mediated knockdown of TXNDC5 significantly attenuated key pathological features of LIRI, including vascular leakage, inflammatory infiltration, and apoptosis. These results not only confirm TXNDC5′s pathogenic role but also highlight its therapeutic potential. Although our *in vivo* knockdown strategy targeted the entire lung, the congruent findings from *in vitro* endothelial-specific manipulations and *in vivo* expression profiling establish pulmonary endothelial TXNDC5 as a central mediator of LIRI. In this study, although we utilized HUVECs as the primary *in vitro* model owing to their well-established stability and applicability for endothelial mechanistic studies [41,42], we acknowledge that differences may exist compared to PMVECs. It is noteworthy, however, that the core findings regarding TXNDC5 upregulation and its functional consequences on the HSP90/eNOS axis and barrier integrity were consistently recapitulated in primary rat PMVECs, strengthening the relevance of our mechanistic insights to LIRI.

The present study also reveals that TXNDC5 activation in LIRI is associated with enhanced inflammatory responses and oxidative stress. Although previous reports present conflicting data on TXNDC5 depletion effects—showing reduced cytokine production in LPS-challenged mice [43] but unaltered responses in bleomycin models [34] —our findings delineate a more precise mechanism. Specifically, TXNDC5 knockdown significantly decreased ICAM-1 and MDA levels while leaving IL-6/IL-10 unchanged, indicating its predominant regulation of leukocyte adhesion and oxidative damage rather than cytokine signaling. This study confirms significant MPO elevation in LIRI, consistent with its role as a marker of neutrophil infiltration and oxidative stress [44]. The observed decrease in MPO and ICAM-1 upon TXNDC5 knockdown likely results from the abrogation of barrier disruption and consequent neutrophil extravasation and adhesion. This specificity aligns with our proteomic data, highlighting enrichment in neutrophil adhesion, NETosis, and stress-response pathways. Notably, emerging evidence positions TXNDC5 as an inflammatory sensor, evidenced by its siRNA-mediated inhibition attenuating arthritis inflammation [13], further supporting our mechanistic observations in LIRI.

Furthermore, we demonstrate that TXNDC5-mediated aggravation of LIRI involves activation of the NF-κB pathway. The anti-inflammatory agent DEX for neutrophil modulation and interferon programming [45,46], while protecting against injury, showed a synergistic effect when combined with TXNDC5 knockdown, including inhibition of the NF-κB pathway and improvement of barrier function. A recent single-cell sequencing and external data study found that DEX mainly inhibits neutrophil infiltration and degranulation in the lungs and blood [47]. This suggests that DEX may act, in part, by mitigating the TXNDC5-driven inflammatory response and underscores the potential of combining TXNDC5 inhibition with anti-inflammatory strategies for superior therapeutic outcomes.

### The ATF6-TXNDC5-eNOS axis: A maladaptive ER stress response

Emerging evidence indicates that ER stress impairs vascular function through eNOS suppression, although the underlying mechanisms remain unclear [48,49]. Our study provides mechanistic clarity by demonstrating that (1) OGD/R-induced ER stress compromises HUVEC barrier integrity via TXNDC5 upregulation and (2) ER stress inhibitors restore barrier function by reducing TXNDC5 expression. The observed inverse correlation between TXNDC5 and eNOS levels suggests that ER stress mediates endothelial dysfunction through a TXNDC5-eNOS regulatory axis.

Although the ATF6-TXNDC5 pathway has been well characterized in pulmonary fibrosis [34] and kidney fibrosis [50], its role in acute vascular injury settings such as I/R remained unexplored. We identify ATF6 as the predominant UPR branch mediating I/R-induced TXNDC5 upregulation in HUVECs. A previous study found that SGLT2 inhibitors could ameliorate vascular calcification through blocking ERS-dependent TXNDC5 upregulation and promoting subsequent Runx2 proteasomal degradation [16]. Although transient ER stress is adaptive, sustained activation promotes apoptosis [[51], [52], [53]]. Our findings suggest TXNDC5 inhibition may provide targeted therapy for LIRI by selectively modulating maladaptive ER stress responses, offering potential advantages over broad-spectrum ER stress inhibitors. Whereas previous research established TXNDC5′s involvement in chronic disease processes, our work identifies its critical role in acute sterile inflammation and endothelial barrier dysfunction during LIRI. The demonstration of an ATF6-TXNDC5-eNOS/HSP90 axis represents a previously unrecognized mechanism underlying pulmonary endothelial dysfunction in this acute setting. Future studies examining post-injury therapeutic interventions, such as administration of ATF6 inhibitors after reperfusion, will be crucial to validate the translational potential of targeting TXNDC5 in clinical LIRI. Although Ceapin-A7 is a specific ATF6 inhibitor, potential off-target effects remain a consideration. Future genetic studies will be valuable to confirm these findings.

### Clinical translation: TXNDC5 as a potential predictive biomarker

In our prior multi-cohort investigation, we examined potential protein markers for LIRI in patients following CPB surgery [16]. Building upon our aforementioned study, we found that TXNDC5 levels were consistently elevated postoperatively across multiple time points in Cohort 1 through proteomic analysis. This finding was validated in Cohorts 2 and 3, where plasma TXNDC5 concentrations showed significant increases in patients with LIRI and correlated with clinical indicators of lung dysfunction (prolonged intubation time and reduced PF ratio). The developed nomogram integrating TXNDC5 with CPB duration and massive red blood cell transfusion demonstrates immediate clinical utility, enabling risk stratification 24 h earlier than radiographic changes. This early-warning capability offers a critical window for therapeutic intervention. Given TXNDC5′s endothelial-specific expression and plasma stability (24 h post-CPB), it could overcome limitations of existing biomarkers and demonstrate significant potential as an early-warning indicator for clinical LIRI. Our findings position TXNDC5 as a potential therapeutic target worthy of further investigation. Clinicians now combine bedside data with new biomarkers to track lung injury more precisely, spotting severe ARDS early, acting faster, and judging prognosis better [9].

We acknowledge the association of TXNDC5 with chronic fibrotic pathologies [[12], [13], [14], [15]]. However, our findings reveal its distinct role in the acute-phase response, characterized by rapid upregulation immediately post-CPB (T1). This temporal specificity differentiates its acute predictive function from chronic fibrotic expression. Most importantly, our comparative analysis with sepsis-ARDS demonstrates that TXNDC5 elevation shows specificity for “sterile” inflammatory injury in LIRI, rather than “infectious” inflammation. Therefore, TXNDC5 represents a promising context-specific early biomarker for LIRI risk stratification in relevant clinical settings such as CPB. Future studies are needed to validate this specificity across a broader range of ARDS etiologies, including trauma.

### Strengths and limitations

This study offers comprehensive mechanistic and translational insights into TXNDC5′s role in LIRI through an integrated multidisciplinary approach. The parallel investigation in well-characterized animal and cell models, and three independent clinical cohorts, strengthens the biological plausibility of our findings. Particularly noteworthy is the tentative demonstration of TXNDC5′s dual role as both a mechanistic mediator (via ATF6-dependent ER stress signaling) and a clinically potential biomarker (with superior AUC and NRI compared to conventional clinical parameters). The development of a nomogram incorporating TXNDC5 levels with key clinical parameters represents a significant advance toward personalized risk assessment in perioperative lung injury. Although our internally validated model provides a foundational framework for LIRI risk stratification, its translation into clinical practice is a future goal that hinges on successful external validation in independent, multi-center cohorts.

While mechanistically robust, our study has some translational limitations. First, although rat LIRI models recapitulate key pathological features, species-specific differences in CPB physiology and ER stress responses may affect clinical extrapolation. Second, the efficacy of TXNDC5 inhibition in established ARDS remains to be validated in late-intervention models. Third, the current AAV-mediated approach lacks cell-type specificity. Endothelial-specific delivery systems (e.g., endothelial-specific nanoparticles or siRNA formulations) are needed to minimize off-target effects of therapeutic targeting in future studies. Finally, TXNDC5 is also implicated in chronic fibrotic diseases, which may limit its absolute specificity as a biomarker for LIRI. However, our data indicate that its primary predictive value lies in the acute phase following I/R, a context pathophysiologically distinct from chronic fibrotic processes. Importantly, the lack of significant TXNDC5 upregulation in septic ARDS underscores its more specific role in sterile inflammatory injury, reinforcing its context-specific relevance for LIRI. These limitations highlight important gaps between mechanistic understanding and clinical application that will require addressing in subsequent translational studies.

## Conclusion

Our study establishes TXNDC5 as a central regulator of LIRI, driving PEB damage via ATF6-dependent ER stress activation and eNOS/HSP90 suppression. The dual utility of TXNDC5—as a key pathological effector and a quantifiable clinical marker—provides new opportunities for targeted therapeutic strategies and risk stratification.

## CRediT authorship contribution statement

**Yu Wang:** Writing – original draft, Data curation, Conceptualization, Methodology, Visualization, Funding acquisition. **Pu Chen:** Writing – original draft, Data curation, Conceptualization, Methodology, Visualization. **Shiqian Huang:** Writing – original draft, Data curation, Conceptualization, Methodology, Visualization. **Lin Chen:** Methodology, Resources. **Yangyang Ge:** Visualization. **Heng Gu:** Methodology. **Yangqi Chu:** Data curation. **Yiyi Yang:** Resources, Data curation. **Yun Lin:** Writing – review & editing, Supervision. **Shanglong Yao:** Writing – review & editing, Supervision, Project administration, Funding acquisition.

## Ethics approval

The clinical study protocol was approved by the Institutional Review Board of Union Hospital, Tongji Medical College (approval no. 20200518), and written informed consent was obtained from all participants. All animal experiments were conducted in accordance with the ARRIVE guidelines and approved by the Institutional Animal Care and Use Committee of Tongji Medical College (IACUC no. 2916).

## Funding

This research was supported by Hubei Technological Innovation Special Fund (2019ACA167), the National Natural Science Foundation of China (82002100), the Bethune Medical Foundation (grant number: bnmr-2021-002) and the China International Medical Foundation (grant number: Z-2017-24-2421).

## Declaration of competing interest

The authors declare that they have no known competing financial interests or personal relationships that could have appeared to influence the work reported in this paper.
