# Supplementary material for: Endoplasmic reticulum stress exacerbates ischemia-reperfusion-induced pulmonary endothelial barrier dysfunction by activating TXNDC5
Source: J Adv Res. 2025 Dec 26;87:773–91. doi: 10.1016/j.jare.2025.12.039 (PMC13539253; doi:10.1016/j.jare.2025.12.039)
Supplement: Supplementary file 1 [file mmc1.docx]

**Appendix A:**

**Endoplasmic Reticulum Stress Exacerbates Ischemia-Reperfusion-Induced Pulmonary Endothelial Barrier Dysfunction by Activating TXNDC5**

**Yu Wang et al.**

[Fig. S1: KEGG pathway enrichment analysis of differentially expressed proteins in LIRI	3](#_Toc213524043)

[Fig. S2. The expression of TXNDC5 in primary rat PMVEC and alveolar epithelial cells (AT1 and AT2) after OGD/R	4](#_Toc213524044)

[Fig. S3. OGD/R induces endothelial dysfunction in primary rat pulmonary microvascular endothelial cells (PMVECs)	5](#_Toc213524045)

[Fig. S4. Validation of TXNDC5 knockdown efficiency in Human umbilical vein endothelial cells (HUVECs)	6](#_Toc213524046)

[Fig. S5. TXNDC5 knockdown showed minimal effect on p-eNOS (Thr495)	7](#_Toc213524047)

[Fig. S6. TXNDC5 knockdown in rat lungs was confirmed by qPCR and Western blot analysis	8](#_Toc213524048)

[Fig. S7. TXNDC5 knockdown significantly reduced inflammatory cell counts compared to the LIRI group	9](#_Toc213524049)

[Fig. S8. TXNDC5 knockdown significantly reduced the oxidative stress marker MDA compared to the LIRI group	10](#_Toc213524050)

[Fig. S9. ChIP-seq profiling identifies a significant ATF6 binding peak at the TXNDC5 proximal promoter.	11](#_Toc213524051)

[Fig. S10. Ceapin-A7 rescues the OGD/R-induced downregulation of VE-cadherin and ZO-1 protein expression as shown by Western blot analysis	12](#_Toc213524052)

[Fig. S11. Sequence homology analysis of human and rat TXNDC5 protein	13](#_Toc213524053)

[Fig. S12. Heatmap of protein expression in LIRI patients compared to Non-LIRI patients at T1 and T2. TXNDC5 was highlighted by the red box.	14](#_Toc213524054)

[Table S1. Univariable logistic regression coefficients for clinical risk factors and CPB-LIRI, along with their eligibility for subsequent screening	15](#_Toc213524055)

[Table S2. Multivariable logistic regression coefficients for clinical risk factors and CPB-LIRI, along with their eligibility for subsequent screening	16](#_Toc213524056)

[Table S3. Nomogram-based prediction model for LIRI	17](#_Toc213524057)

[Method S1: Establishment protocol for clinical cohorts	18](#_Toc213524058)

[Method S2: The protocol for the TXNDC5 prediction model	20](#_Toc213524059)

[Method S3: The number of animals for different experimental procedures Animal experimental protocols	25](#_Toc213524060)

[Method S4: Animal experimental protocols for intratracheal injection of AAV in rats	28](#_Toc213524061)

[Method S5: Proteomic detection and analysis of rat lung tissue	29](#_Toc213524062)

[Method S6: Apoptosis analysis	32](#_Toc213524063)

[Method S7: Protein Analysis	33](#_Toc213524064)

[Method S8: Real-Time quantitative PCR	35](#_Toc213524065)

[Method S9: Chromatin Immunoprecipitation followed by Sequencing (ChIP-Seq) and ChIP-qPCR	36](#_Toc213524066)

# Fig. S1: KEGG pathway enrichment analysis of differentially expressed proteins in LIRI

The analysis revealed neutrophil extracellular trap formation, cell adhesion molecules, and apoptosis as the most significantly altered pathways (P < 0.05). These pathways are associated with key pathological processes in LIRI, including inflammation, endothelial dysfunction, and cell death.

# Fig. S2. The expression of TXNDC5 in primary rat PMVEC and alveolar epithelial cells (AT1 and AT2) after OGD/R

rPMVEC: Rat pulmonary microvascular endothelial cells; rAT1: Alveolar epithelial type I cell; rAT2: Alveolar epithelial type II cell; OGD/R: oxygen-glucose deprivation/reoxygenation.

# Fig. S3. OGD/R induces endothelial dysfunction in primary rat pulmonary microvascular endothelial cells (PMVECs)

(A) Representative phase-contrast image showing the typical cobblestone morphology of primary rat PMVECs under normal culture conditions. (B) Apoptosis analysis by flow cytometry revealing significantly increased apoptotic rates in PMVECs subjected to OGD/R. (C-F) Western blot analysis demonstrating downregulated protein expression of HSP90, eNOS and TXNDC5 in PMVECs following OGD/R injury. (G) Transendothelial electrical resistance (TEER) measurements showing impaired barrier function in PMVECs after OGD/R. Data are presented as mean ± SEM; *p < 0.05, **p < 0.01 vs. control group (Student's t-test). All experiments were performed with at least three biological replicates.

# Fig. S4. Validation of TXNDC5 knockdown efficiency in Human umbilical vein endothelial cells (HUVECs)

(A) Representative Western blot images and quantitative analysis showing TXNDC5 protein expression following knockdown. (B) qPCR analysis confirming the reduction of TXNDC5 mRNA levels. Data are presented as mean ± SEM (n = 3); *p < 0.05, ***p < 0.001 vs. shNC group (Student's t-test). All experiments were performed with at least three biological replicates

# Fig. S5. TXNDC5 knockdown showed minimal effect on p-eNOS (Thr495)

(A) Representative Western blot images and (B) quantitative analysis of p-eNOS (Thr495) protein levels in endothelial cells subjected to OGD/R with or without TXNDC5 knockdown. The suppressive phosphorylation at eNOS Thr495 was not affected by either OGD/R injury or TXNDC5 silencing. Data are presented as mean ± SEM. All experiments were performed with at least three biological replicates.

# Fig. S6. TXNDC5 knockdown in rat lungs was confirmed by qPCR and Western blot analysis

(A) TXNDC5 mRNA levels in whole lung homogenates from Sprague-Dawley (SD) rats were significantly reduced as measured by qPCR. (B) Representative Western blot images and (C) quantitative analysis demonstrating decreased TXNDC5 protein expression following knockdown. Data are presented as mean ± SEM; **p < 0.01, ****p < 0.0001 vs. sham group (Student's t-test). The experiment was performed with five biological replicates.

# Fig. S7. TXNDC5 knockdown significantly reduced inflammatory cell counts compared to the LIRI group

The amount of inflammatory cells (sum of 10 400x field) in the Sham, ShNC+Sham, ShNC+LIRI, ShTxndc5+LIRI group. Statistical significance was determined using one-way ANOVA with Tukey’s post hoc test. ns: *P*≥0.05; **P*<0.05. The experiment was performed with three biological replicates.

# Fig. S8. TXNDC5 knockdown significantly reduced the oxidative stress marker MDA compared to the LIRI group

Quantification of MDA levels in lung homogenates. Statistical significance was determined using one-way ANOVA with Tukey’s post hoc test. ns: *P*≥0.05; **P*<0.05. The experiment was performed with three biological replicates.

# Fig. S9. ChIP-seq profiling identifies a significant ATF6 binding peak at the TXNDC5 proximal promoter.

# Fig. S10. Ceapin-A7 rescues the OGD/R-induced downregulation of VE-cadherin and ZO-1 protein expression as shown by Western blot analysis

Western blot analysis shows that (A, B) ZO-1 and (A, C) VE-cadherin protein levels, which were reduced following OGD/R injury, and significantly restored upon Ceapin-A7 treatment. Note: data are presented as mean ± SEM. Statistical significance was determined using one-way ANOVA with Tukey’s post hoc test. ns: *P*≥0.05; **P*<0.05; ***P*<0.01; ****P*<0.001. All experiments were performed with at least four biological replicates.

# Fig. S11. Sequence homology analysis of human and rat TXNDC5 protein

Homology comparison of TXNDC5 nucleic acid between humans and rats. The comparison was performed using the National Center for Biotechnology Information (NCBI) database. The E value represents the probability of a random match, where smaller values denote higher sequence similarity. The red line in the figure denotes the matching sequences, with the majority of sequences demonstrating homology.

# Fig. S12. Heatmap of protein expression in LIRI patients compared to Non-LIRI patients at T1 and T2. TXNDC5 was highlighted by the red box.

# Table S1. Univariable logistic regression coefficients for clinical risk factors and CPB-LIRI, along with their eligibility for subsequent screening

|  | Estimate | Std. Error | z value | P value | next analyses |
| --- | --- | --- | --- | --- | --- |
| Smoking | 0.549 | 0.374 | 1.468 | 0.142 | out |
| Alcohol | 0.3 | 0.389 | 0.772 | 0.44 | out |
| Hypertension | 0.354 | 0.345 | 1.028 | 0.304 | out |
| Diabetes | 1.329 | 0.821 | 1.62 | 0.105 | out |
| Stroke | 0.405 | 0.454 | 0.893 | 0.372 | out |
| Hematocrit | -0.024 | 0.032 | -0.742 | 0.458 | out |
| hemoglobin | -0.006 | 0.009 | -0.687 | 0.492 | out |
| White blood cell count | 0.005 | 0.118 | 0.045 | 0.964 | out |
| albumin | -0.01 | 0.066 | -0.156 | 0.876 | out |
| ALT | 0.017 | 0.009 | 1.759 | 0.079 | out |
| AST | 0.012 | 0.01 | 1.252 | 0.211 | out |
| creatinine | 0.005 | 0.005 | 0.985 | 0.325 | out |
| Input volume | 0 | 0 | -0.56 | 0.576 | out |
| **Anesthesia time** | **0.004** | **0.002** | **1.967** | **0.049** | **in** |
| CPB time | 0.012 | 0.004 | 3.405 | 0.001 | in |
| Electric defibrillation | 0.641 | 0.362 | 1.771 | 0.077 | out |
| **Ultrafiltration volume** | **0** | **0** | **2.141** | **0.032** | **in** |
| Precharge fluid volume | -0.001 | 0.001 | -0.882 | 0.378 | out |
| Additional fluid volume | 0 | 0.001 | -0.795 | 0.426 | out |
| **Cardioprotective fluid volume** | **0.001** | **0** | **2.041** | **0.041** | **in** |
| Urine volume | 0 | 0 | -0.39 | 0.697 | out |
| Transfusion reaction | 0.773 | 0.576 | 1.342 | 0.18 | out |
| **Volume of RBC transfusion** | **0.232** | **0.069** | **3.373** | **0.001** | **in** |
| **Volume of plasma transfusion** | **0.002** | **0.001** | **4.147** | **0** | **in** |
| **Volume of platelet transfusion** | **0.567** | **0.238** | **2.382** | **0.017** | **in** |
| **MRBC** | **1.702** | **0.37** | **4.599** | **0** | **in** |
| ALT, Alanine aminotransferase; AST, Glutamic oxaloacetic transaminase; CPB, cardiopulmonary bypass; RBC, red blood cells; MRBC, massive transfusion of red blood cells | | | | | |

# Table S2. Multivariable logistic regression coefficients for clinical risk factors and CPB-LIRI, along with their eligibility for subsequent screening

|  | Estimate | Std. Error | z value | P value | next analyses |
| --- | --- | --- | --- | --- | --- |
| CPB time | 0.015 | 0.007 | 2.252 | 0.024 | in |
| Anesthesia time | -0.005 | 0.003 | -1.588 | 0.112 | out |
| Ultrafiltration volume | 0 | 0 | 1.393 | 0.164 | out |
| Cardioprotective fluid volume | 0 | 0 | -0.168 | 0.867 | out |
| Volume of RBC transfusion | -0.089 | 0.111 | -0.805 | 0.421 | out |
| Volume of plasma transfusion | 0.365 | 0.24 | 1.522 | 0.128 | out |
| Volume of platelet transfusion | 0.001 | 0.001 | 1.926 | 0.054 | out |
| MRBC | 1.462 | 0.581 | 2.516 | 0.012 | in |
| CPB, cardiopulmonary bypass; RBC, red blood cells; MRBC, massive transfusion of red blood cells | | | | | |

# Table S3. Nomogram-based prediction model for LIRI

|  | Estimate | 95% CI | P value |
| --- | --- | --- | --- |
| AUC |  |  |  |
| 3-fold | 0.810 | 0.803, 0.816 |  |
| 5-fold | 0.805 | 0.795, 0.815 |  |
| 10-fold | 0.811 | 0.796, 0.826 |  |
| NRI | 0.457 | 0.266, 0.648 | < 0.05 |
| IDI | 0.114 | 0.060, 0.168 | < 0.05 |
| CI, confidence interval; NRI, net reclassification improvement; IDI, integrated discrimination improvement | | | |

# Method S1: Establishment protocol for clinical cohorts

A nested case‒control study design was chosen, discovery-based biomarker screening was used in Cohort 1 (April 2021 to July 2021, n = 150), and validation strategies were used in Cohort 2 and 3 (August 2021 to July 2022, n = 375; January 2023 to March 2023, n = 124). Twenty pairs of case‒controls in Cohort 1 were selected for perioperative proteomics profile determination and screening of differentially expressed proteins (DEPs) at multiple time points. Quantitative analysis and validation of TXNDC5, as well as training and internal validation of a TXNDC5-based prediction model, were carried out within Cohort 2, 3.

All adult subjects over 18 years old undergoing elective CPB for valves with or without coronary artery surgery were eligible for the study. The exclusion criteria included the following: (I) patients who refuse to sign the informed consent form or the attending physician refuses to allow the patient to join the study; (II) nonelective surgery (surgery at a nonelective time or emergency surgery); (III) preoperative pulmonary insufficiency, pulmonary hypertension and pulmonary inflammation; (IV) the absence of any specimen and clinical data; and (V) patients who had a failed operation, needed extracorporeal membrane oxygenation support or underwent CPB operation again within 3 days after operation. Patients who developed LIRI in the first 3 days after CPB were placed into the LIRI group. The flow diagram showing the recruiting criteria is as follows.

In Cohort 1 for the proteomics analyses, once the case was determined, an additional patient was matched as a control in the non-LIRI patient group based on the following criteria: same group of surgeons; same type of operation; same gender; and the differences of BMI and age were within ±10% compared with the LIRI patient.

In Cohort 2 and 3, for the development of the prediction model, multiple clinical variables and protein variables were included in the multivariable logistic regression. The sample size for the development of the final multivariable logistic regression should be more than 20 times the number of predictors.

*Flow diagram showing the recruiting criteria.*

Exclusion criteria included the following: (I) patients who refuse to sign the informed consent form or the attending physician refuses the patient to join the study; (II) non elective surgery (surgery at non-elective time or emergency surgery); (III) preoperative pulmonary insufficiency, pulmonary hypertension and pulmonary inflammation; (IV) the absence of any specimen and clinical data; (V) patients who failed the operation, needed extracorporeal membrane oxygenation support or underwent CPB operation again within 3 days after operation.

# Method S2: The protocol for the TXNDC5 prediction model

**(1) Clinical data and outcome data collection**

Potential perioperative ARDS clinical risk factors that have been reported in previous studies were collected by trained investigators[1-5]. The clinical variables were collected from preoperation to the end of CPB operation, only representative indicators for prediction before the onset of ARDS were considered [6]. The following data were included: (1) demographic information and preoperative complications; (2) preoperative laboratory values; (3) CPB surgery-related variables, including intraoperative infusion; anesthesia time; CPB time; electric defibrillation, ultrafiltration volume, precharge fluid volume, additional fluid volume, cardioprotective fluid volume; (4) blood transfusion: volume of red blood cell (RBC) transfusion; volume of plasma transfusion; volume of platelet transfusion; and massive transfusion of red blood cells (MRBC).

The candidate clinical risk variables for the prediction of LIRI and correction of confounding factors included the following parts:

**1. demographic information and preoperative complications**

1) Sex: Male; Female

2) Age: Unit: years

3) BMI: Body Mass Index: Weight (kg) divided by height (m) squared, Unit: kg/m^2^

4) Smoking: Preoperative smoking history

5) Alcohol: Preoperative drinking history

6) Hypertension: Preoperative history of hypertension

7) Diabetes: Preoperative history of diabetes

8) Stroke: Preoperative history of stroke

**2. preoperative laboratory values**

1) Hematocrit: Unit: %

2) Hemoglobin: Unit: g/L

3) White blood cell count: Unit: ×10^9^/L

4) Albumin: Unit: g/L

5) Alanine aminotransferase: Unit: U/L

6) Glutamic oxaloacetic transaminase: Unit: U/L

7) Creatinine: Unit: μmoI/L

**3. CPB surgery related variables**

1) Surgery type: Complex: Multivalvular surgery, or valvular surgery combined with coronary artery surgery; Simple: Single valvular surgery

2) Input volume: Intraoperative intravenous infusion volume; Unit: ml

3) Anesthesia time: Time from anesthetic administration to the end of operation; Unit: min

4) CPB time: Time from start to end of cardiopulmonary bypass; Unit: min

5) Electric defibrillation: Electric defibrillation during cardiopulmonary bypass

6) Ultrafiltration volume: Unit: ml

7) Precharge fluid volume (PFV): Unit: ml

8) Additional fluid volume (AFV): Unit: ml

9) Cardioprotective fluid volume (CFV): Unit: ml

10) Urine: Intraoperative urine volume; Unit: ml

**4. blood transfusion (during operation)**

1) Transfusion reaction: Blood transfusion reaction refers to the adverse reaction caused by the transfusion of blood or its products or the infusion equipment used during or after the transfusion

2) Transfusion of red blood cells (RBC): Total unit of red blood cells transfused during operation

3) Plasma transfusion: Total unit of plasma during operation

4) Platelet transfusion: Total unit of platelet during operation

5) Massive RBC: Transfusion of > 3 units of red blood cells intraoperatively

**(2)** **The detailed diagnosis method of the primary outcome**

The primary outcome variable was the development of ARDS within 3 days after CPB (LIRI). Based on the Berlin definition [7], LIRI was independently confirmed by 2 physicians who reviewed the medical records. Chest X-rays of these patients were performed daily or twice per day. Blood gases were examined every 4–6 h. Respiratory failure in patients with LIRI was not fully explained by cardiac failure or fluid overload and was evaluated twice from echocardiography, clinical symptoms and other indicators.

Specific diagnostic information is as follows:

**1. Main diagnostic indicators**

The primary outcome variable was the development of LIRI within 3 days after CPB. Based on the Berlin definition, LIRI was described as the following: (1) hypoxemia, occurring within 1 week of a known clinical insult or new or worsening respiratory symptoms; (2) chest image with bilateral opacities that cannot be fully explained by pleural effusions, atelectasis, or nodules; (3) respiratory failure that are not fully explained by cardiac failure or fluid overload; (4) Also, the oxygenation index (PaO_2_/FiO_2_) shall be at least lower than 300 mmHg in patients receiving positive end-expiratory pressure (PEEP) or continuous positive airway pressure (CPAP) ≥ 5 cmH_2_O.

**2. Specific diagnostic details**

**1) Onset time:** occurring within 3 days after CPB.

**2) Chest image:**

① **Chest imaging frequency:** Chest X-rays of these patients were performed daily or twice per day and examined by senior cardiac surgeons and the intensivist during the ICU stay;

② **Change characteristics:** new ground glass density shadow (GGO), consolidation (CO), patch shadow, strip shadow and bilateral lung texture thickening after operation;

③ **Distribution characteristics:** scattered and uniform distribution of both lungs, and less central or peripheral distribution.

**3) Respiratory failure not fully explained by cardiac failure or fluid overload:**

not fully explained by cardiac failure or fluid overload, and meet at least 3 of the following;

① If echocardiography is performed, it shows normal systolic function (ejection fraction >45%) and diastolic function (e:e ′<15);

② Or according to the function of the valve examined by transesophageal echocardiography after operation;

③ Central venous pressure <14 mmhg;

④ No systemic hypertension or volume overload;

⑤ No brain natriuretic peptide level increased more than 1.5 times the upper limit of normal;

⑥ No clinical symptoms of heart failure were recorded in the daily progress or consultation records (unable to lie flat and sit in an upright position, acute onset, pink foam sputum, etc.).

**4) Oxygenation index (PaO_2_/FiO_2_):**

① Frequency: Blood gases were examined every 4–6 h, or according to the patient's condition change and the cardiac surgeon's advice.

② Duration: PaO_2_/FiO_2_ ratio <300 mmHg and PEEP/CPAP ≥ 5 cmH_2_O for at least 3~4 consecutive hours.

**(3) Parsimonious model in Cohort 2 and 3**

Concerning missing data, our prospective study design ensured complete capture of all predefined clinical variables. We can confirm no missing data exist for any clinical parameters analyzed. The 19 excluded patients for (inclusion/exclusion criteria IV) the absence of any specimen and clinical data were removed specifically due to missing plasma samples required for protein analysis, not due to any absence of clinical data.

Regarding confounder adjustment, we addressed this through our modeling strategy. The clinical data from Cohort 2 and 3 were preliminarily tested using univariable logistic regression algorithm. Then, the clinical variables with P < 0.05 were entered into a stepwise logistic regression algorithm (SLR) to screen clinical predictors (P<0.05 in SLR of clinical factors).

For the collection time of samples, the earlier collection of samples in the development of LIRI may deduce the biological signals of LIRI in a timely manner and enhance efforts to prevent and intervene [8]. Considering that time point T1 was immediately after CPB injury and before the occurrence of LIRI, TXNDC5 levels at T1 may be more conducive to the prediction of the early stages of LIRI.

Finally, the TXNDC5 concentration and the screened clinical predictors were combined to construct the combined TXNDC5-based prediction model. The model was evaluated for model differentiation ability.

All assessments of predictors were conducted blind to the case‒control status. All computations were conducted in the R environment, version R version 4·2·0 (April 2022). The results with P ≤ 0·05 were considered statistically significant.

**References:**

1.	Kor DJ, Lingineni RK, Gajic O, Park PK, Blum JM, Hou PC, et al. Predicting risk of postoperative lung injury in high-risk surgical patients: a multicenter cohort study. Anesthesiology. 2014; 120: 1168-81.

2.	Gajic O, Dabbagh O, Park PK, Adesanya A, Chang SY, Hou P, et al. Early identification of patients at risk of acute lung injury: evaluation of lung injury prediction score in a multicenter cohort study. Am J Resp Crit Care. 2011; 183: 462-70.

3.	Mazzeffi M, Kassa W, Gammie J, Tanaka K, Roman P, Zhan M, et al. Preoperative Aspirin Use and Lung Injury After Aortic Valve Replacement Surgery: A Retrospective Cohort Study. Anesth Analg. 2015; 121: 271-7.

4.	Chen SW, Chang CH, Chu PH, Chen TH, Wu VC, Huang YK, et al. Risk factor analysis of postoperative acute respiratory distress syndrome in valvular heart surgery. J Crit Care. 2016; 31: 139-43.

5.	Xie J, Liu L, Yang Y, Yu W, Li M, Yu K, et al. A modified acute respiratory distress syndrome prediction score: a multicenter cohort study in China. J Thorac Dis. 2018; 10: 5764-73.

6.	Trillo-Alvarez C, Cartin-Ceba R, Kor DJ, Kojicic M, Kashyap R, Thakur S, et al. Acute lung injury prediction score: derivation and validation in a population-based sample. Eur Respir J. 2011; 37: 604-9.

7.	Ranieri VM, Rubenfeld GD, Thompson BT, Ferguson ND, Caldwell E, Fan E, et al. Acute respiratory distress syndrome: the Berlin Definition. Jama-J Am Med Assoc. 2012; 307: 2526-33.

8.	Wen XP, Zhang YZ, Wan QQ. Non-targeted proteomics of acute respiratory distress syndrome: clinical and research applications. Proteome Sci. 2021; 19: 5.

# Method S3: The number of animals for different experimental procedures Animal experimental protocols

**1. Animals**

Fifty male Sprague Dawley rats (Beijing Vital River Laboratory Animal Technology Co., Ltd.; Certificate number SYXK2021-0011), weighted 300 ± 20 g, were housed in animal experiment center of the SPF environment (light/dark cycles 12/12 h, the temperature of 22–24 °C, the humidity of 60–65%) with free access to food and water. All experimental protocols were approved by the Animal Care and Use Committee of Tongji Medical College of Huazhong University of Science and Technology, and were conducted in accordance with the Chinese Animal Research Guidelines.

**2. Grouping and Animal Handling**

A subset of the rats (n=74) was randomly divided into 2 groups: sham group and LIRI group. Randomization of grouping used random numbers generated by SAS software. The number of animals for different experimental procedures in sham and LIRI groups is summarized in Table M1.

| **Table M1.** Number of animals for different experimental procedures for the sham and LIRI groups. | | | |
| --- | --- | --- | --- |
| Groups | Sham | LIRI | Total |
| Hematoxylin and eosin | 3 | 3 | 6 |
| MPO activity detection | (3) | (3) |  |
| TMT proteomics detection | 3 | 3 | 6 |
| Western blotting | 6 | LIRI 2 h/6 h/24 h: n = 6 each (Total N = 18) | 24 |
| Transmission electron microscopy | 3 | 3 | 6 |
| Tunel staining | 5 | 5 | 10 |
| Immunofluorescence | (3) | (3) |  |
| Single cell transcriptome analysis | 3 | 3 | 6 |
| W/D ratio | 4 | 4 | 8 |
| Evans blue–albumin extravasation assay | 4 | 4 | 8 |
| Note: Sham, sham group; LIRI, lung ischemia-reperfusion injury group. The same rats were used for assessment of Western blotting and MPO activity; The same rats were used for Hematoxylin and eosin, and Immunofluorescence. Number in parentheses means the same rats were used as the upper row. | | | |

An additional cluster (94 rats) were sequentially randomly assigned to four groups: sham, sham + shNC, LIRI + shNC, LIRI + shTxndc5 group. Randomization of grouping used random numbers generated by SAS software. The number of animals for different experimental procedures in sham, sham + shNC, LIRI + shNC, LIRI + shTxndc5 groups is summarized in Table M2.

| **Table M2.** Number of animals for different experimental procedures for the sham, sham + shNC, LIRI + shNC, LIRI + shTxndc5 groups. | | | | | |
| --- | --- | --- | --- | --- | --- |
| Groups | sham | sham+shNC | LIRI+shNC | LIRI+shTxndc5 | Total |
| Frozen sections | 5 | 5 (ShTxndc5+Sham) |  |  | 10 |
| fluorescence-activated cell sorting (FACS) | 3 | 3 (ShTxndc5+ShNC) |  |  | 6 |
| Hematoxylin and eosin | 3 | 3 | 3 | 3 | 12 |
| Tunel staining | 3 | 3 | 3 | 3 | 12 |
| Transmission electron microscopy | 3 | 3 | 3 | 3 | 12 |
| Western blotting | 3 | 3 | 3 | 3 | 12 |
| Inflammatory markers (ELISA) | (3) | (3) | (3) | (3) |  |
| Oxidative stress markers detection | (3) | (3) | (3) | (3) |  |
| Immunofluorescence |  |  | 3 | 3 | 6 |
| W/D ratio | 3 | 3 | 3 | 3 | 12 |
| Evans blue–albumin extravasation assay | 3 | 3 | 3 | 3 | 12 |
| Note: Sham, sham group; LIRI, lung ischemia-reperfusion injury group. The same rat was used for assessment of Western blotting, ELISA, and Oxidative stress markers. Number in parentheses means the same rats were used as the upper row. | | | | | |

An additional cluster (36 rats) were sequentially randomly assigned to four groups: Sham, LIRI, LIRI+DEX, LIRI+ShTxndc5+DEX group. Randomization of grouping used random numbers generated by SAS software. The number of animals for different experimental procedures is summarized in Table M3.

| **Table M3.** Number of animals for different experimental procedures for the Sham, LIRI, LIRI+DEX, LIRI+ShTxndc5+DEX groups. | | | | | |
| --- | --- | --- | --- | --- | --- |
| Groups | Sham | LIRI | LIRI+DEX | LIRI+ShTxndc5+DEX | Total |
| Western blotting | 3 | 3 | 3 | 3 | 12 |
| W/D ratio | 3 | 3 | 3 | 3 | 12 |
| Evans blue–albumin extravasation assay | 3 | 3 | 3 | 3 | 12 |
| Note: Sham, sham group; LIRI, lung ischemia-reperfusion injury group. The same rat was used for assessment of Western blotting, ELISA, and Oxidative stress markers. Number in parentheses means the same rats were used as the upper row. | | | | | |

# Method S4: Animal experimental protocols for intratracheal injection of AAV in rats

① Virus preparation: Prior to commencing the experiment, the virus was thawed on ice after removal from the -80°C freezer.

② Virus injection: SD rats were randomly assigned to one of four groups: Sham, ShNC+Sham, ShNC+LIRI, and ShTxndc5+LIRI, using a randomization protocol. Following the administration of anesthesia with 2% pentobarbital sodium (0.05 mg/g), the rats in each group were labeled (to prevent label shedding during the two-week viral infection period, rats from different groups were housed separately).

Subsequent to skin disinfection, the trachea was accessed via a midline cervical incision. An insulin needle was used to aspirate the thawed virus, which was then rapidly injected into the trachea through the flat ring-shaped cartilage. Immediately following the injection, an injection of 0.5 mL of air into the syringe was performed, and this procedure was repeated three times to minimize residual AAV virus. Subsequently, the surgical plate was rotated to ensure even distribution of the virus throughout the lungs. Once the skin was sutured, the rat was transferred to a warm blanket with the head and chest elevated to facilitate unobstructed respiration and enhance viral infection in the lower lungs.

③ Virus infection and detection: After regaining consciousness, the SD rats were returned to their feeding cages and provided with water and feed. Following a two-week period to ensure uniform lung infection, the rats were euthanized to facilitate the collection of lung samples. These samples were processed into frozen lung sections, which were subsequently examined for viral infection using green fluorescent markers. Western blot (WB) analysis was conducted on the lung tissue homogenate to assess the efficacy of TXNDC5 knockdown post-viral infection.

# Method S5: Proteomic detection and analysis of rat lung tissue

Sample Treatment Samples were added to the lysis buffer (1.5% SDS/100 mM Tris-Cl) and mixed uniformly for tissue homogenization. After centrifugation at 16,000 × g for 15 minutes, the supernatant was collected. Proteins in the solution were precipitated using acetone, followed by the addition of the resolubilization buffer (8 M Urea/100 mM Tris-HCl, pH=8.5) to the protein pellet for protein resolubilization. After determining the protein concentration of each sample using the BCA method, 200 µg of protein from each sample was transferred to a new tube and adjusted to 100 µl with 1% SDC. An equal volume of double-distilled water was added to reduce the SDC concentration to 0.5% in each tube. TCEP and CAA were added for disulfide bond reduction and cysteine-SH alkylation reaction at 37°C for 1 hour. After disulfide bond reduction and alkylation, 100 mM Tris-HCl solution was added to the samples, followed by the addition of 4 µg trypsin and incubation with shaking overnight at 37°C. The enzymatic digestion was terminated with 1% TFA, and the supernatant was collected for SDB-RPS desalting column desalting. The desalted peptides were dried using a Speed Vac and stored at -20°C.

TMT Labeling Prior to LC-MS/MS analysis, lung tissue proteins were labeled with TMT. Equal amounts of samples were used for TMT labeling. After completion, a desalting step (Sep-Pak C18) was performed, followed by drying and fractionation, ultimately combining into 15 fractions for detection.

LC-MS/MS Analysis Mass spectrometry data were acquired using a Q Exactive HF-X mass spectrometer coupled with an UltiMate 3000 RSLCnano liquid chromatography system. The peptide samples were dissolved in the loading buffer, inhaled by the automatic sampler and combined to the analysis column (75 μm*25cm, C18, 3 μm particle size, 100 Å pore size, Thermo) for separation. The two mobile phases (mobile phase A: 0.1% formic acid, and mobile phase B: 0.1% formic acid, 80% acetonitrile) were used to establish a 90 min separation gradient. A constant flow rate was set at 300 nL/min. For the data acquisition in data-independent acquisition (DIA) mode, each scan cycle includes one full scan MS1 (R = 60 K, AGC = 3e6, Max IT = 30 ms, Scan range = 350–1250 m/z) and 40 subsequent MS2 scans (R = 15 K, AGC = 1e5, max IT = 50 ms). High energy collision dissociation (HCD) collision energy was set to 28.

Database Search and Comparison The search software employed was MaxQuant (V1.6.6), with the Andromeda algorithm for database searching. The database used for searching was the Uniprot Rat proteome reference database (2019-04-21, containing 29,947 protein sequences). The main search parameters were as follows: Project type: TMT; Variable modifications: Oxidation (M), Acetyl (Protein N-term); Fixed modifications: Carbamidomethyl ©; Enzyme: Trypsin/P; The mass tolerance for the primary MS match was set at 20 ppm for the initial search and 4.5 ppm for the main search; the mass tolerance for the secondary MS match was set at 20 ppm. The search results were filtered at a 1% FDR (false discovery rate) for both precursor ions and protein levels, removing reverse database proteins, contaminant proteins, and proteins with only one modified peptide. The remaining identifications were used for subsequent analysis.

Protein Data Cleaning and DEPs Selection Protein quantification data were log_2_-transformed to conform to a normal distribution, followed by normalization (subtracting the median). Partial missing data were treated with the random tail imputation method, using half of the minimum value for imputation. DEPs selection criteria were FC greater than 1.2 times or less than 1/1.2 times; and a P value < 0.05 for intergroup t-tests.

GO and KEGG Enrichment Analysis The R language package “clusterProfiler (4.4.4)” was used to search the authoritative international databases Uniprot, GO, and KEGG for functional annotation and enrichment analysis of the identified DEPs. The pathway enrichment test used the hypergeometric distribution test, and a corrected P value (FDR correction) less than 0.05 was considered significant. All functional annotation analyses were sorted according to the E-ratio.

For the analyses of various functional annotation, the DEPs were assigned to the categories of different “biological processes (BP)”, “cellular components (CC)”, and “molecular functions (MF)”. Here, we defined:

N = number of human proteins annotated by at least one term

n = number of human proteins annotated by term t

M = number of the DEPs annotated by at least one term

m = number of the DEPs annotated by term t

Then, the E-ratio was calculated, and the p value was computed with the hypergeometric distribution as below:

$$E-ratio=\frac{\left( m/M \right)}{\left( n/N \right)}$$

$$P value=\sum_{m'=m}^{n} \frac{\left( \begin{matrix} M \\ m' \end{matrix} \right)\left( \begin{matrix} N-M \\ n-m' \end{matrix} \right)}{\left( \begin{matrix} N \\ n \end{matrix} \right)}, \left( E-ratio>1 \right)$$

In this study, only statistically enriched GO terms (adjusted P value by FDR <0.05) and KEGG pathways (adjusted P value by FDR <0.05) were considered. The enriched items were sorted according to the E-ratio.

# Method S6: Apoptosis analysis

(1) Tunel staining

Detection of apoptosis in lung tissue in vivo using the Tunel assay kit (Elabscience, Cat. No: E-CK-A322). Paraffin sections of 4 μm thick lung tissue were stained according to the instructions after deparaffinization and permeabilization. Sections were scanned and photographed under a VS120 laser scanning confocal microscope (Olympus, Tokyo, Japan). Tunel-positive cells (nuclei stained red) were found in 5 random and non-overlapping areas. Apoptotic index was calculated as the ratio of Tunel-positive cells to all cells.

(2) Flow cytometry

HUVECs and primary PMVECs in each group were incubated with fluorescein FITC-labeled annexin V (Annexin-V-FITC) and PI using an apoptosis kit according to the manufacturer’s instructions (Elabscience, Cat. No: E-CK-A211). And HUVECs transfected with the AAV virus were incubated with fluorescein APC-labeled annexin V (Annexin-V-APC) and 7-AAD (Elabscience, Cat. No: E-CK-A230). After 15 min of incubation in the dark at room temperature, cell death was analyzed by flow cytometry (CytoFLEX; Beckman Coulter). Survival cells were defined as double-negative, early apoptosis were defined as Annexin-V-FITC positive and PI/7-AAD negative, late apoptosis was defined as double-positive, and death cells were defined as PI/7-AAD positive and Annexin-V-FITC negative.

# Method S7: Protein Analysis

(1) Western blot

Lung tissues and HUVECs were homogenized in RIPA lysis buffer (Beyotime Biotechnology, Shanghai, China) containing the protease inhibitor and phosphatase inhibitors. Protein concentrations were determined using Bicinchoninic acid (CoWin Biosciences, Inc., Beijing, China). Proteins (20–50 μg) were boiled, separated on appropriate percentage SDS-PAGE (7.5%, 10% and 12.5%), and transferred onto polyvinylidene fluoride membranes (PVDF) membranes. After 1 h blocking in sealing fluid (Epizyme Biomedical Technology Co., Ltd, Shanghai, China), the membranes were incubated with primary antibodies for TXNDC5 (1:15000, Cat#: 19834-1-AP, Proteintech, USA), Bip (1:1000, Cat#: A23453, ABclonal, China), ATF6 (1:1000, Cat#: ab37149, abcam, USA), IRE1 (1:1000, Cat#: A21021, ABclonal), PERK (1:1000, Cat#3192, Cell Signaling Technology, USA), HSP90 (1:2000, Cat#: 13171-1-AP, Proteintech), t-eNOS (1:1000, Cat#: AF0096, Affinity), Phospho-eNOS (Thr495) (1:1000, Cat#: AF3248, Affinity), Phospho-eNOS (Ser1177) (1:1000, Cat#: AF3247, Affinity), ZO-1 (WB:1:5000, IF:1:200, Cat#: 21773-1-AP, Proteintech), VE Cadherin (WB:1:500, IF:1:100, Cat#: A0734, ABclonal), Claudin 5 (WB:1:1000, IF:1:500, Cat#: A25830, ABclonal), NF-κB p65 (WB:1:1000, Cat#: 8242, Cell Signaling Technology), Phospho-NF-κB p65 (Ser536) ( WB:1:1000, Cat#: 3036, Cell Signaling Technology), IκB alpha (WB:1:1000, Cat#: T55026, Abmart), Phospho-IκB alpha (Ser32/Ser36) ( WB:1:1000, Cat#: TP56280, Abmart), or β-actin (1:50000, Cat#: AC026, ABclonal) at 4°C overnight, and subsequently with peroxidase-conjugated secondary antibodies for 1 h at room temperature. Proteins were then visualized using electrochemiluminescence reagents and quantified using the NIH Image J software.

(2) Immunofluorescent analyse

Freshly prepared lung OCT sections were incubated with 0.2% Triton X-100 in PBS for 15 min followed by 5% bovine serum albumin for 30 min at room temperature. The slides were then incubated with rabbit anti-TXNDC5 (1:100, Cat#: 19834-1-AP, Proteintech, USA), anti-ZO-1 (1:200, Cat#: 21773-1-AP, Proteintech), anti-VE Cadherin (1:100, Cat#: A0734, ABclonal), anti-Claudin 5 (1:500, Cat#: A25830, ABclonal) respectively at 4°C overnight. After washing, the slides were incubated with FITC-labeled goat anti-rabbit or goat anti-mouse secondary antibody respectively for 60 min at room temperature. Slides were counterstained with DAPI, cover-slipped, and fluorescent images captured using a Nikon A1R Confocal Microscope (Tokyo, Japan).

Quantitative Immunofluorescence Analysis of Junctional Proteins:

(1) Digital slide processing was performed using QuPath 5.1 and OpenSlide software to convert whole-slide images and separate fluorescence channels.

(2) AI-assisted annotation was applied to delineate the overall tissue analysis area under pathologist supervision. For each slide, three random fields were selected, and the average value was used for quantitative analysis.

(3) AI algorithms and ImageJ software quantified the numbers of 0, 1+, 2+, and 3+ positive cells and calculated the H-SCORE, as well as the mean optical density (mean OD) for each fluorescence channel within the defined tissue region.

The H-SCORE was calculated using the following formula:

H-SCORE = [1 × (% of cells with intensity score 1) + 2 × (% of cells with intensity score 2) + 3 × (% of cells with intensity score 3)].

# Method S8: Real-Time quantitative PCR

Real-time reverse transcriptase-polymerase chain reaction (RT-PCR) analysis was performed (Bio-Rad, Maastricht, The Netherlands) to determine the transcript levels of the following genes:

*Txndc5*:	Forward primer	AGCTGCATGTTTCTCAAGGC

Reverse primer	TGCAGTCAACCTTGCCAATC

*Actb*:	Forward primer	TTGAACACGGCATTGTCACC

Reverse primer	TTTTCACGGTTGGCCTTAGG

*TXNDC5*:	Forward primer	AAACTGGATGCTGCAGACAC

Reverse primer	ATAAAGTGGTCGCCTTGTGC

*ACTB:*	Forward primer	TCGTGCGTGACATTAAGGAG

Reverse primer	TTGCCAATGGTGATGACCTG

The primer sequences of these genes were determined by the NCBI software analysis of Primer BLAST. The details of the sequences and thermal cycling conditions were according to the standard protocol. Data were acquired and analyzed with the IQ5 software (Bio-Rad, Maastricht, The Netherlands).

# Method S9: Chromatin Immunoprecipitation followed by Sequencing (ChIP-Seq) and ChIP-qPCR

Chromatin immunoprecipitation assays were performed according to the previously described by Wuhan IGENEBOOK Biotechnology Co. Ltd. Briefly, HUVECs in 100mm petri dishes were washed twice in cold PBS buffer and cross-linked with 1% formaldehyde for 10 minutes at room temperature and then quenched by addition of glycine (125 mmol/L final concentration). Afterwards, samples were lysed with 50mM Tris-HCl (pH 8.0), 10mM EDTA, 1%SDS, 1×protease inhibitor cocktail, and chromatins were obtained on ice. Chromatins were sonicated to get soluble sheared chromatin (average DNA length of 200–500 bp). 20ul chromatin was saved at –20°C for input DNA, and 100ul chromatin was used for immunoprecipitation by anti-ATF6 antibody-ChIP Grade (ab227830, abcam). 10 μg of antibody was used in immunoprecipitation reactions at 4 °C overnight. The next day, 30 μL of protein G beads (10004D, Thermofisher) was added and the samples were further incubated for 3 h. Bound material was then eluted from the beads in 300 μL of elution buffer (100 mM NaHCO3, 1% SDS), treated first with RNase A (final concentration 8 μg/mL) during 6 h at 65°C and then with proteinase K (final concentration 345 μg/mL) overnight at 45°C. Immunoprecipitated DNA was used to construct sequencing libraries following the protocol provided by the I NEXTFLEX® ChIP-Seq Library Prep Kit for Illumina® Sequencing (NOVA-5143-02, Bioo Scientific) and sequenced on Illumina Novaseq 6000 with PE 150 method. DNA Fragments are sequenced and analyzed by Wuhan IGENEBOOK Biotechnology Co. Ltd.

In addition, a portion of the DNA was used for real-time reverse transcriptase-polymerase chain reaction (RT-PCR) analysis. The primer sequences are as follows:

Forward primer: TGAATTCAACCGCCTCTTGC

Reverse primer: CGGGGTGACATTTGGACTC
